# Single-nucleus transcriptomics of IDH1- and TP53-mutant glioma stem cells displays diversified commitment on invasive cancer progenitors

**DOI:** 10.1038/s41598-022-23646-3

**Published:** 2022-11-08

**Authors:** Valeriia Gulaia, Mikhail Shmelev, Aleksander Romanishin, Nikita Shved, Vladislav Farniev, Nikolay Goncharov, Arthur Biktimirov, Irene Lisa Vargas, Konstantin Khodosevich, Alexander Kagansky, Vadim Kumeiko

**Affiliations:** 1 Institute of Life Sciences and Biomedicine, Medical Center, Far Eastern Federal University, Vladivostok, 690922 Russia; 2 A.V. Zhirmunsky National Scientific Center of Marine Biology, FEB RAS, Vladivostok, 690041 Russia; 3 School of Life Sciences, Immanuel Kant Baltic Federal University, Kaliningrad, 236041 Russia; 4 Biotech Research & Innovation Centre (BRIC), The Faculty of Health and Medical Sciences, University of Copenhagen, 2200 Copenhagen, Denmark

**Keywords:** Cancer, Computational biology and bioinformatics, Molecular biology, Molecular medicine

## Abstract

Glioma is a devastating brain tumor with a high mortality rate attributed to the glioma stem cells (GSCs) possessing high plasticity. Marker mutations in isocitrate dehydrogenase type 1 (*IDH1*) and tumor protein 53 (*TP53*) are frequent in gliomas and impact the cell fate decisions. Understanding the GSC heterogeneity within *IDH1-* and *TP53-* mutant tumors may elucidate possible treatment targets. Here, we performed single-nucleus transcriptomics of mutant and wild-type glioma samples sorted for Sox2 stem cell marker. For the first time the rare subpopulations of Sox2 + *IDH1-* and *TP53*-mutant GSCs were characterized. In general, GSCs contained the heterogeneity root subpopulation resembling active neural stem cells capable of asymmetric division to quiescent and transit amplifying cell branches. Specifically, double-mutant GSCs revealed the commitment on highly invasive oligodendrocyte- and astroglia-like progenitors. Additionally, double-mutant GSCs displayed upregulated markers of collagen synthesis, altered lipogenesis and high migration, while wild-type GSCs expressed genes related to ATP production. Wild-type GSC root population was highly heterogeneous and lacked the signature marker expression, thus glioblastoma treatment should emphasize on establishing differentiation protocol directed against residual GSCs. For the more differentiated *IDH1*- and *TP53*-mutant gliomas we suggest therapeutic targeting of migration molecules, such as CD44.

## Introduction

Glioma is the most common primary brain tumor and represents the least curable malignancy, which treatment failure is rooted, on the one hand, in cell heterogeneity and, on the other, in cell plasticity. In turn, cell heterogeneity/plasticity features could depend on driver glioma mutations highlighted by World Health Organization Guidelines from 2016 for glioma classification^1^.

Mutations in isocitrate dehydrogenase 1/2 (*IDH1/2*) and tumor protein 53 (*TP53*) constitute a molecular base for astrocytoma diagnostics. Furthermore, 41% of patients carrying any type of p53 mutations causing proto-oncogenic activity (gain-of-function mutation or dominant-negative effect (GOF/DN)), also have mutant *IDH1*^2^. The widely recognized *TP53* GOF/DNs are located at R175, R248 and R273 residues and facilitate the expansion of glioma stem cells (GSCs)^3,4^. At the same time, GSCs constitute a dynamic population characterized by a plastic intermediate stemness state adoptable by the majority of the cancer stem cell (CSC) progeny^5^. In other words, differentiated glioma cells are capable of reversing the differentiation process and recapitulating the stemness properties pertained to multi- or unipotent cells^6^. GSCs populations express such markers, as CD133, Sox2, Oct4, CD44, CD15, Nanog, Nestin, etc.^7^, while only Sox2 and Oct4 drive stem cell pluripotency^8^. Sox2 + is a master regulator of embryonic stem cell pluripotency^9^ and reverses the differentiation process in normal somatic cells^10^. Meanwhile, Sox2 has been multiply shown to be critical in glioma relapse and therapeutic resistance^11,12^.

GSCs from IDH-wild type glioblastomas are characterized to express *PTPRZ1*^6^, Sox2 and Nanog^13^, CD9, *PROM1* (CD133), *PDGFRA* and *OLIG2*^14^. Proliferative cell population from IDH-mutant gliomas has neural progenitor cell-like profile expressing CD24^5,15^. However, there is no characterization of GSC population from *IDH*- and *TP53*-mutant gliomas, therefore we still lack a systematic understanding of the driver mutation impact on the GSC fate, differentiation status and plasticity. We hypothesize, that the combination of missense mutations in *IDH1* and *TP53* would lead to certain GSC phenotypes distinctive to astrocytoma and secondary glioblastoma.

The aim of the study was to reveal Sox2 + GSCs marker genes raised due to the missense mutations in *IDH1* and *TP53*. To unify the impact of background mutational load we decided to compare *IDH1*- and *TP53*-mutant glioma samples to glioma samples having the wild-type genes. The overall mutational load in gliomas does depend on glioma grade, but regardless of the *IDH* mutation^16,17^. So, we reasoned that the inclusion of two high-grade and one low-grade sample per each subgroup would unify the mutational load.

Mutations in *IDH1* and *TP53* skewed GSCs towards low proliferation and committed to differentiation phenotype, which was replenished by the high migration activity. Additionally, mutant GSCs had vulnerability points in (i) enhanced collagen synthesis, (ii) insufficiency of endogenic lipogenesis, (iii) CD44 expression. The revealed points can be clinically tackled on the protein/metabolic level.

## Results

### Sox2 staining distinguish tumor CSCs

**Figure 1:**
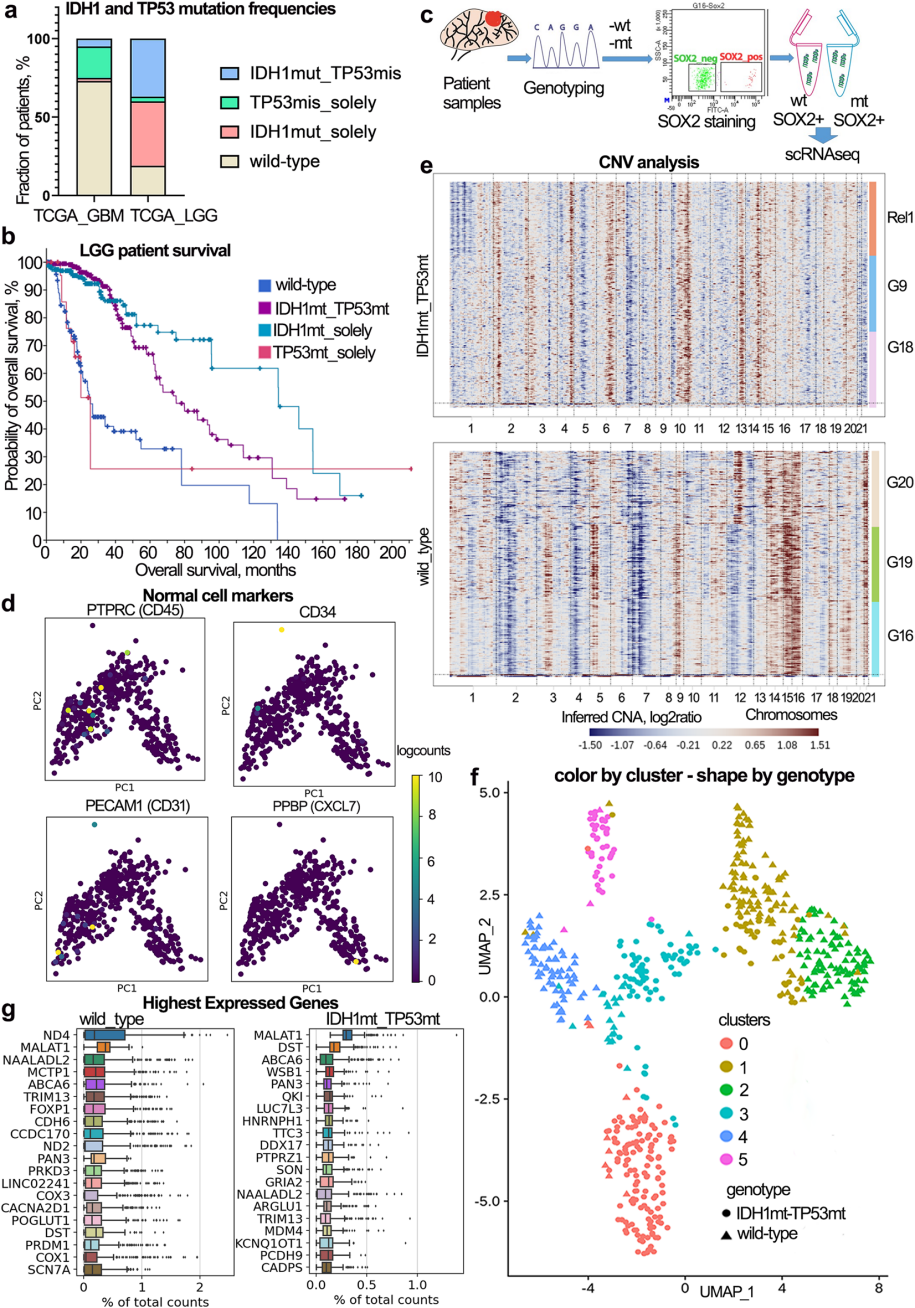
Sample selection and filtering GSC data. (**A**) Barplot displaying the frequencies (in %) of missense mutations in IDH1 and TP53 genes for high-grade gliomas (TCGA_GBM) and low-grade gliomas (TCGA_LGG) within the TCGA dataset. The color annotated the respective mutational profile – IDH1mut-TP53mis (missense mutations in both IDH1 and TP53 genes), TP53mis_solely (missense mutation only in TP53), IDH1mut_solely (missense mutation only in IDH1), and wild-type (wild-type version of both IDH1 and TP53 genes). (**B**) The overall survival probability plot of patients with low-grade gliomas depending on mutations in IDH1 and TP53. The color legend on right side annotated the patient mutational profile as described above. Data were taken from the cBioPortal. (**C**) Schematic of research workflow used to obtain glioma stem cell profiles – primary patient samples partitioned for genotyping and snap freezing, then grouped by wt- and mt-samples. Wt- and mt-samples were lysed to obtain single nuclei, stained for Sox2, and sorted for the snRNAseq (Smart-seq2 protocol). (**D**) Expression of the characteristic hematopoietic genes in all dataset. (**E**) Chromosomal rearrangements occurring in sequenced cells. Cells (rows) contained a duplication/amplification (red), deletion (blue), or wild-type variant (uncolored). Cells were labeled by tumor samples (side bar) and chromosome name (bottom bar). (**F**) The highest expressed gene plots showing filtered raw counts for highly expressed genes in sequenced cells for wt- and mt-samples. (**G**) UMAP plot showing the clustering of GSCs colored by cluster and shaped by genotype.

To strengthen the clinical relevance, we quired TCGA data for the *IDH1* and *TP53* missense mutation frequencies. Low-grade glioma (LGG) patients had combination of missense mutations in *IDH1* and *TP53* in 37% of cases, while glioblastoma (GBM) patients – only in 5% of cases (Fig. 1A). The overall survival (OS) probability negatively correlated with the presence of *TP53* missense mutation in LGG patients (Fig. 1B). Thus, LGG patients bearing mutations in *IDH1* and *TP53* together had median OS of 75 months, while patients having *IDH1* mutation solely survived 2 times longer – 134 months (Table S3). On the contrary, GBM patient OS correlated with *IDH1* mutation, so both *IDH1* single mutant and *IDH1*/*TP53*-double mutant patients had higher probability of survival, compared to wild-type patients (Fig. S1).

To comprehensively interrogate inter-tumoral heterogeneity in stem cell populations from *IDH1*- and *TP53*-mutant gliomas, we profiled snap-frozen tumor samples from 6 patients (Fig. 1C; Table 1) using full-length single nucleus RNA sequencing (snRNA-seq). To focus on malignant nuclei, we sorted them by both 7-AAD and neural stem cell (NSC)/GSC marker Sox2 (Fig. S2A). The gate for Sox2-negative population was based on background staining with IgG-isotype control antibody, and we placed Sox2-positive gate further leaving the blank space. However, due to the difference in staining between samples, we acquired both Sox2 + ^dim^ and Sox2 + ^high^ populations (Fig. S2B). We profiled Sox2 + cells from *IDH1*- and *TP53*-mutant samples (here and below mt-samples, mt-group, mt-GSCs, mt-cells) versus *IDH1*- and *TP53* wild-type samples (here and below wt-samples, wt-group, wt-GSCs, wt-cells). In total, 576 Sox2 + nuclei were profiled for mt- and wt-samples (Fig. 1C) with average detection of 1900 features and 180,000 counts per cell (Fig. S3A). Normal cells were excluded on the basis of specific hematopoietic and interstitial markers (Fig. 1D, Fig. S3B), the rest were subjected to chromosomal evaluation.

**Table 1:** Patient’s samples collected.

Sample name	Diagnosis, grade	Gender	Mutations, CNVs*	Purpose of use
G9	Secondary glioblastoma, G4	Male	-IDH1 R132H -TP53 R273C -13-amplification	Transcriptomics
G16	Glioblastoma, G4	Male	- wild-type -2p-deletion, 7p-deletion, 15-amplification	Transcriptomics
G18	Anaplastic astrocytoma, G3	Male	-IDH1 R132H -TP53 R248Q -13-amplification	Transcriptomics
G19	Fibrillary protoplasmic astrocytoma, G2	Female	-wild-type -2p-deletion, 5q-amplification, 7p-deletion, 15-amplification	Transcriptomics
G20	Glioblastoma multiforme, G4	Female	-wild-type -12q-amplification	Transcriptomics
Rel1	Diffuse oligodendroglioma, G2	Female	-IDH1 R132H -1p-deletion	Transcriptomics
BT32	Anaplastic astrocytoma, G3	Male	-IDH1 R132H	Cell culture
BT40	Glioblastoma multiforme, G4	Female	-wild-type	Cell culture
BT29	Anaplastic astrocytoma, G3	Male	-IDH1 R132H -TP53 R248Q	Cell culture

*based on CNV-analysis for single-cell data from Fig. 1C.

Typical chromosomal alterations for high grade gliomas include amplification of chromosome 7 and 19, and loss of chromosome 10^18^, while *IDH1*-mutant gliomas more frequently loose 1p/19q and gain chromosomes 8 and 10^19,20^. Almost all the remaining cells exhibited big chromosomal rearrangements, though mt-samples (G18, G9, Rel1) display less pronounced chromosomal alterations compared to wild-type ones (G16, G19, G20). Wt-samples exhibited clear loss of chromosomes 2, 4, 7 and 12 and amplification of chromosomes 14, 15 and 16. Mt-samples displayed loss of 7q and 10q and amplification of 6q, 10q, 13 and 14q (Fig. 1E, Fig. S3C). Basing on chromosomal rearrangements data, we can conclude that the majority of remaining cells were of glioma origin.

The unsupervised clustering of remaining cells (570 nuclei) was performed using UMAP and formed six large cell clusters (Fig. 1F).

Preliminary analysis of the highest expressed genes revealed overexpression of *MDM4*, a controller of TP53 function, in the wt-group, but not in mt-samples, which can indicate the need for mutant TP53 functioning for the survival benefits. Additionally, we observed the high expression of stem cell markers in both mt- and wt-samples, including *FOXP1*, *POGLUT1*, and *PTPRZ1* (Fig. 1G).

Thus, we suggest the Sox2 staining was efficient for (i) separating malignant glioma cells from hematopoietic, interstitial cells, and oligodendrocytes, (ii) enriching for stem cells. Our data set is relatively small comparing to similar glioma single cell studies^5,6,21–23^, however enrichment for GSCs allows us to look for subtle stem cell populations that typically got shed in the big studies.

### GSCs display quiescent, proliferating and migrating phenotypes

**Figure 2:**
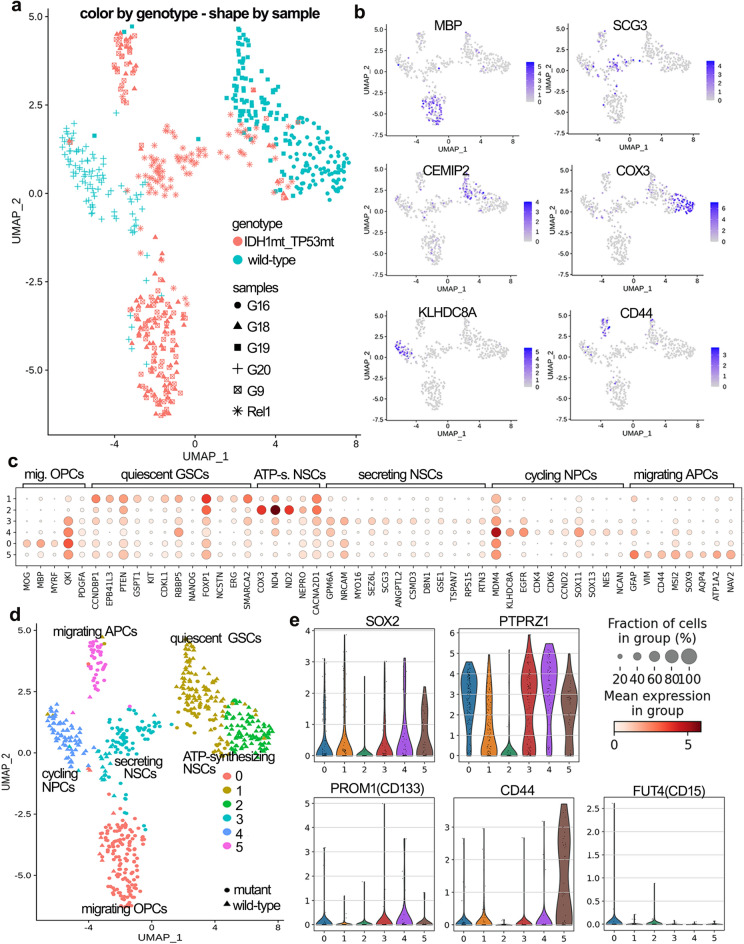
Cell cluster annotation. (**A**) UMAP plot showing the clustering of GSCs colored by genotype and shaped by glioma sample. (**B**) Expression of characteristic cluster-specifying genes overlaid on the 2D-UMAP space. (**C**) Stack circle plot showing expression of genes characterizing 6 UMAP clusters named “migrating OPCs” (mig. OPCs), “quiescent GSCs”, “ATP-synthesizing NSCs” (ATP-s. NSCs), “secreting NSCs”, “cycling NPCs”, “migrating APCs”. (**D**) UMAP clusters colored by clusters and shaped by genotype with annotated cell types. (**E**) Violin plots highlighting the expression of the selected stem cell genes (SOX2, PTPRZ1, PROM1, CD44, FUT4) for UMAP clusters.

The distribution of samples between the clusters was not uniform. Sample G9 and G18 mostly formed cluster 0 and cluster 5, Rel1 and G19 made cluster 1, G16 structured cluster 2, G20 and Rel1 composited cluster 3, and G20 mainly assembled cluster 4. Although, samples G19 and Rel1 are low-grade gliomas (Table 1), we decided to render them for the analysis as they were clustered together with other samples – G20 and G16 (Fig. 2A).

Differential expression analysis between UMAP subpopulations revealed several signature genes defining subgroup features (Fig. 2C). Thus, cluster 0 was characterized by the expression of oligodendrocyte genes, such as *MOG*, *MBP*, *MYRF*, *PLP1* (Fig. S4A, Fig. S4G) implicated in myelin synthesis and stabilization, as well as stem cell and oligodendrocyte progenitor cell (OPC) genes – *QKI*, *FRYL*, *PTGDS*, and *PDGFA*. Additionally, this subpopulation revealed intensive collagen synthesis and bearing the variety of the migration receptors (Fig. S4G, Fig. S8A, Fig. S8B). Accordingly, cluster 0 cells were referred to as “migrating OPCs'' summarizing their phenotypic features – oligodendrocytes expressing stem cell genes, bringing OPCs, and their functional role – migration. Using this logic, we attempted to classify the remained subpopulations.

Cluster 1 was characterized by upregulation of tumor-suppressing genes: *HNF1B*, *CCNDBP1*, *PATJ*, *EPB41L3*, *MARVELD2*, *PTEN* in conjunction with cell-cycle genes – *GSPT1*, *GPR19*, *EAPP*, *KIT*, *CDKL1*, and stem cell markers – *RBBP5*, *NANOG*, *NCSTN*, *ERG*, including quiescent stem cell markers—*FOXP1*, *SMARCA2* (Fig. 2B, C, Fig. S4B, Fig. S4G). Apparently, this population should display protracted cell-cycle elongated by the active tumor-suppressing genes though balanced by the proliferation genes. We suggested these cells displayed long retention in G0, while still being ready to pass the G2/M checkpoint. ccSeurat classifier appointed most of cluster 1 cells to be in the S phase (Fig. S5B), but this classifier was never trained to recognize G0 phase. Therefore, we used ccAF capable of recognizing so called Neural-G0 trained on human NSCs^24^, by which we unraveled that 42% of Cluster 1 cells were in Neural G0 phase and 43% in G1 phase (Fig. S5C).This cluster was named “non-cycling GSCs” or “quiescent GSCs”.

Cluster 2 was named “ATP-synthesizing NSCs”, as it expressed oxidative phosphorylation genes – *COX3*, *ND4*, *ND2*, etc., and neural progenitor markers – *NEPRO*, *CACNA2D1*, *EPHA5*, *SETD5*, *TRIM64B* (Fig. 2C, Fig. S4C, Fig. S4G). Although, oxide phosphorylation genes are intrinsically sourced from the mitochondrial genome and could denote apoptosis, however, they can mark activated oxidative phosphorylation to efficiently produce ATP for rapid cell cycling. Additionally, this subpopulation expressed antiapoptotic genes *MTRNR2L8* and *MTRNR2L1* (Fig. S10D).

Cluster 3 named “secreting NSCs” had neuronal genes, i.e. *NRCAM*, *MYO16*, *SEZ6L*, *DNER*, *NECAB1*, *CSMD3* jointly with the neuroendocrine secretion genes: *RTN1*, *SCG3*, *ANGPTL2* (Fig. 2C, Fig. S4D, Fig. S4G). Additionally, cluster 3 possessed a substantial number of general stem cell and NSC markers – *GPM6A*, *NKD1*, *DBN1*, *GSE1*, *KAT7*, *PPIP5K1*, *TSPAN7*, and transcription regulators, i.e. *RPS1*, *RTN3* (Fig. 2C, Fig. S4G).

Cluster 4 or “cycling neural progenitor cells (NPCs)” expressed receptors for growth factors – *KLHDC8A*, *EGFR*, *MARS1* stabilizing CDK4, in combination with cyclin D2 and its cyclin-dependent kinases and neural progenitor genes – *SOX11*, *SOX13*, *HOXB3*, *AVIL*, *NES*, *ZBED6*, *NCAN*, etc. (Fig. 2C, Fig. S4E, Fig. S4G).

Cluster 5 named “migrating astrocyte-precursor cells (APCs)” possessed markers of premature astrocytes – *GFAP*, *VIM*, *FZD3*, *SOX9*, and mature astrocytes – *AQP4*, *ATP1A2*, as well as stem cell genes – *CD44*, *MSI1*, *MSI2* (Fig. 2C, Fig. S4F). Clearly, this cluster is also responsible for migration and invasion (*CD44*, *NAV2*, *FN1*) on the one hand, and for forming new colonies supported by active Wnt signaling (*FZD3*) on the other.

Thus, *IDH1*- and *TP53*-mutant GSCs (mt-GSCs) mostly displayed invasive APC- and OPC-like phenotypes, while wild-type (wt-GSCs) encompassed cycling, ATP-synthesizing and quiescent subpopulations of neuronal origin. So far, mt-GSCs were more differentiated as they displayed features of oligodendrocytes and early astrocytes (Fig. 2D).

After cluster definition we checked the presence of the classical stemness factors. Surprisingly, *SOX2* was low expressed for all the populations with the exception for cluster 5. This phenomenon could be explained by a higher stability of SOX2 protein over the SOX2 transcript, or by low concentration of SOX2 transcript not allowing to capture it by snRNAseq. The *PROM1* (CD133) and *FUT4* (CD15) were absent, *PTPRZ1* was upregulated almost in all clusters, *CD44* was found in cluster 5 (Fig. 2E). The neuronal lineage marker *CD24*, *POU5F1* (Oct4), and the early progenitor marker *PODXL* were absent, while *NANOG* expressed in the Cluster 1, and *ZFP36L1* was enriched in Cluster 5 (Fig. S10C).

As previously noted, GSCs can be distinguished by the high rate of cell cycle gene expression^23^. The proportion of actively dividing cells entered the G2/M cycle phase was unexpectedly high when assessed with Cyclone^25^ (Fig. S5A). “Migrating OPCs”, “migrating APCs” and “cycling NPCs” comprised large proportion of cells in G2M phase, “ATP-synthesizing NSCs” and “quiescent GSCs” contained the biggest part assigned as cells in S phase (Fig. S5B). On the contrary, ccAF appointed as the most quiescent in our dataset “migrating OPCs” and EGFR + “cycling NPCs” expressing CDK4, CDK6 and CCND2 (Fig. S5C), however we think that the classifier is not adjusted to small population investigation. Additionally, the less differentiated assigned clusters the more the commitment to G1 and S phase but not to mitosis (Fig. S5D) meaning the possible “poised to division” state.

Thus, GSC clusters were described according to their predominant function: (i) migration/invasion, (ii) energy production, (iii) tumor expansion/proliferation. Phenotypically GSCs were characterized by having features of OPCs, NPCs, and APCs.

### GSCs display various differentiation levels and survival features

**Figure 3:**
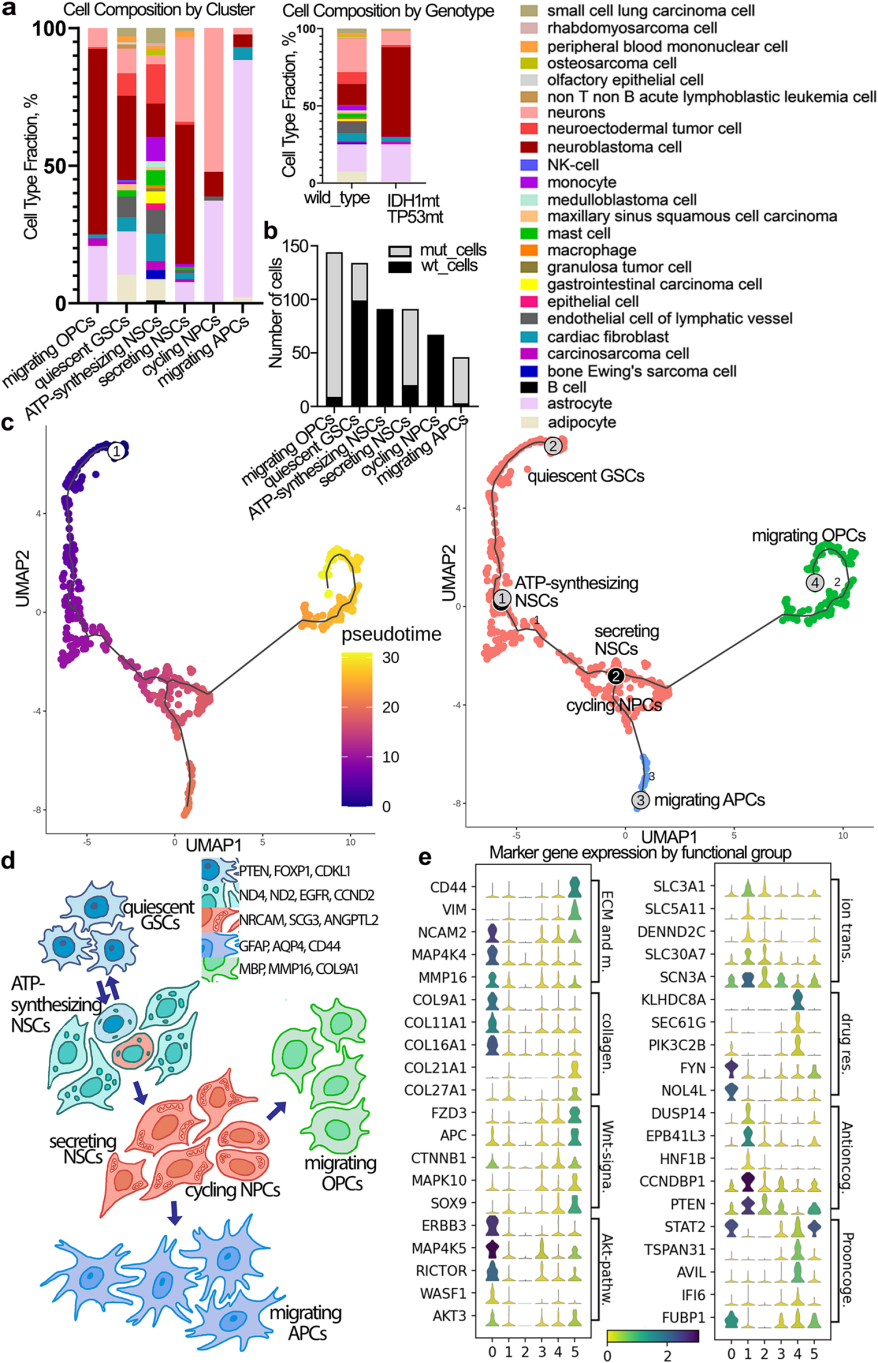
Characterization of the cell cluster features. (**A**) Cell types in % of total cell number assigned to Seurat clusters (left big) and wt- and mt-samples (right small) by scMatch. Color panel describes the assigned cell types (B) Number of wt- (black) and mt- (grey) cells per cluster (**C**) Seurat clusters ordered in accordance with the RNA velocity (the first derivative of newly transcribed RNA and mature RNA) – a differentiation pseudotime by Monocle3. Colored by pseudotime—the shortest distance between a cell and the start of the trajectory (left) and by Seurat clusters (right). (**D**) Model for the GSC hierarchy annotating cell types, their genetic and morphological determinants. Red color indicates cycling cells. ATP-synthesizing NSCs and cycling NPCs have the same color annotating the prevalence of proliferation program in both subpopulations. Left upper panel describes prominent markers for each differentiation step. (**E**) Stack violin plots highlighting marker gene expression grouped as ECM and migration (ECM and m.), collagens (collagen.), Wnt-signalling (Wnt-signa.), Akt-pathway (Akt-pathw.), ion transport (ion trans.), drug resistance (drug res.), antioncogenes (antioncog.), prooncogenes (prooncoge.). Genes grouped by cell annotations (side description) and UMAP clusters (down column bar: 0 – “migrating OPCs”, 1 – “quiescent GSCs”, 2 – “ATP-synthesizing NSCs”, 3 – “secreting NSCs”, 4 – “cycling NPCs”, 5 – “migrating APCs”). The violin shape displays the number of the cells expressing a gene, the continuous color panel defines median expression value of a gene from the absence of expression (yellow) to high expression (dark blue).

To further explore prospective cell types we annotated them using scMatch^26^ by assigning the closest gene expression profiles from the large reference datasets. Mt-samples displayed less heterogeneity, as the major part was assigned to have either astrocyte-like or neuroblastoma-like profile, with a small part identified as neurons (Fig. 3A). At the same time, wt-samples displayed a variety of cell types recognized as normal – adipocytes, cardiac fibroblasts, endothelial cells, or cancerous – gastrointestinal carcinoma, medulloblastoma, small cell lung carcinoma (Fig. 3A). The observed cell type diversity within the wt-samples can be linked to the high rate of morphological and genetical heterogeneity common for high grade gliomas. Additionally, the major part of the heterogeneity was concentrated in the “quiescent GSCs” cluster and in “ATP-synthesizing NSCs” (Fig. 3A, Fig. S6A), that could represent the most stem populations within our dataset.

The most heterogenic population within wt-cells was “ATP-synthesizing NSCs” comprising 21 cell types, and within mt-cells – secreting NSCs possessing 11 cell types. Although, two cell clusters—“quiescent GSCs” and “secreting NSCs” – combined wt- and mt-cells in substantial proportion, the rest populations were more homogeneous – “migrating OPCs/APCs” contained mostly mt-cells, while “cycling NPCs” and “ATP-synthesizing NSCs” encompassed only wt-cells (Fig. 3B, Table S4). The two heterogenic populations were analyzed for the markers labeling wt- and mt-cells to examine the level of intra-cluster heterogeneity. For “secreting NSCs”: wt- and mt-cells expressed 416 and 456 signature genes, respectively; for “quiescent GSCs”: wt-cells had 605 signature genes, and mt-cells expressed 317 genes (Fig. S6B).

To study the cell dynamics trough differentiation processes, we employed Monocle 3^27^. The altogether analysis of mt- and wt-samples revealed, that the root is established in “ATP-synthesizing NSCs” producing, on the one hand, dormant progeny – “quiescent GSCs” inheriting the whole heterogeneity, and, on the other, actively cycling “secreting NSCs” and “cycling NPCs”, subsequently diverged to “migrating OPCs” and “migrating APCs” (Fig. 3C). Thus, the “quiescent GSCs” and “ATP-synthesizing NSCs” clusters represented the most stem populations without firm marker expression, making them elusive for direct targeting. As a result, we adapted the model of adult NSC differentiation suggested by Bond et al.^28^ (Fig. 3D). Considering this putative GSC hierarchy model, described by us earlier^29^, “ATP-synthesizing NSCs” play a role of activated NSCs dividing into “quiescent GSCs” and “cycling/secreting NSCs/NPCs” further differentiating in “migrating OPCs and APCs” (Fig. 3C, D).

Next, we analyzed the pathways, that GSCs could use for proliferation, migration/invasion, therapy evasion and niche construction. “Migrating OPCs” expressed a variety of migratory genes such as *TNS3*, *DSCAM*, *NAV1*, *ADAM33*, *KAZN*, *MAP4K4*, etc., matrix metalloprotease *MMP16*, and *XYLT1* forming extracellular matrix (ECM) (Fig. 3E, Fig. S8B), produced several collagen types, e.g. *COL9A1*, *COL9A2*, *COL9A3*, *COL11A1*, *COL16A1* (Fig. S8A). Similarly, “migrating APCs” also expressed *CD44* and *CRYAB* promoting invasion^30^ (Fig. S8B), and upregulated collagens XXI and XXVII (Fig. S8A).

Among genes supporting growth signaling, “migrating APCs” displayed enhanced level of Wnt messengers (*FZD3*, *SOX9*, *APC*, etc.), and “migrating OPCs” revealed upregulated Akt pathway (*ERBB3*, *ERBB4*, *RICTOR*, *AKT3*) (Fig. 3E, Fig. S9A, Fig. S9B). Additionally, “migrating APCs” had increased expression of ion channels (Fig. S10A) and antioncogenes (Fig. S10B). The observed discrepancy with antioncogene expression can be explained by the presence of residual anticancer mechanisms. On the other hand, upregulation of ion channels could lead to drug resistance.

Additionally, to overcome the size of our dataset we performed the aligning of the identified Seurat clusters on two big studies spanning IDH1mt-TP53mt samples—GSE89567 and wt-samples—GSM3828672. For the mutant cohort we preselected only double-mutant samples with both IDH1 and TP53 mutations (Table S5) and then chose only cells with Sox2 expression above 0 (Fig. S7A). Unfortunately, we found only 4 out of 6 Seurat clusters, moreover, most wt-samples were assigned to be EGFR + “cycling NPCs”, and mt-samples were almost all appointed as “secreting NSCs” (Fig. S7B). Our inability to find the rest of the populations in the confirming dataset could be due to vanishing rare populations in big studies, especially considering that we have found only two most cycling populations (Fig. S5B). However, we also need to admit that the small dataset could give stochastic features as significant due to individual patient variability.

Thus, we suggest the treatment approach based on enforcing the remained anticancer mechanisms and modulating ECM structural compounds (collagens). Additionally, we observed cell populations concentrating heterogeneity features, which can be considered as a main source of glioma plasticity. However, the high marker heterogeneity precludes finding a target therapy, prompting us to suggest the approaches forcing stem cell differentiation (e.g. retinoic acid therapy).

### Mutant GSCs have differentiated and low proliferative phenotype

**Figure 4:**
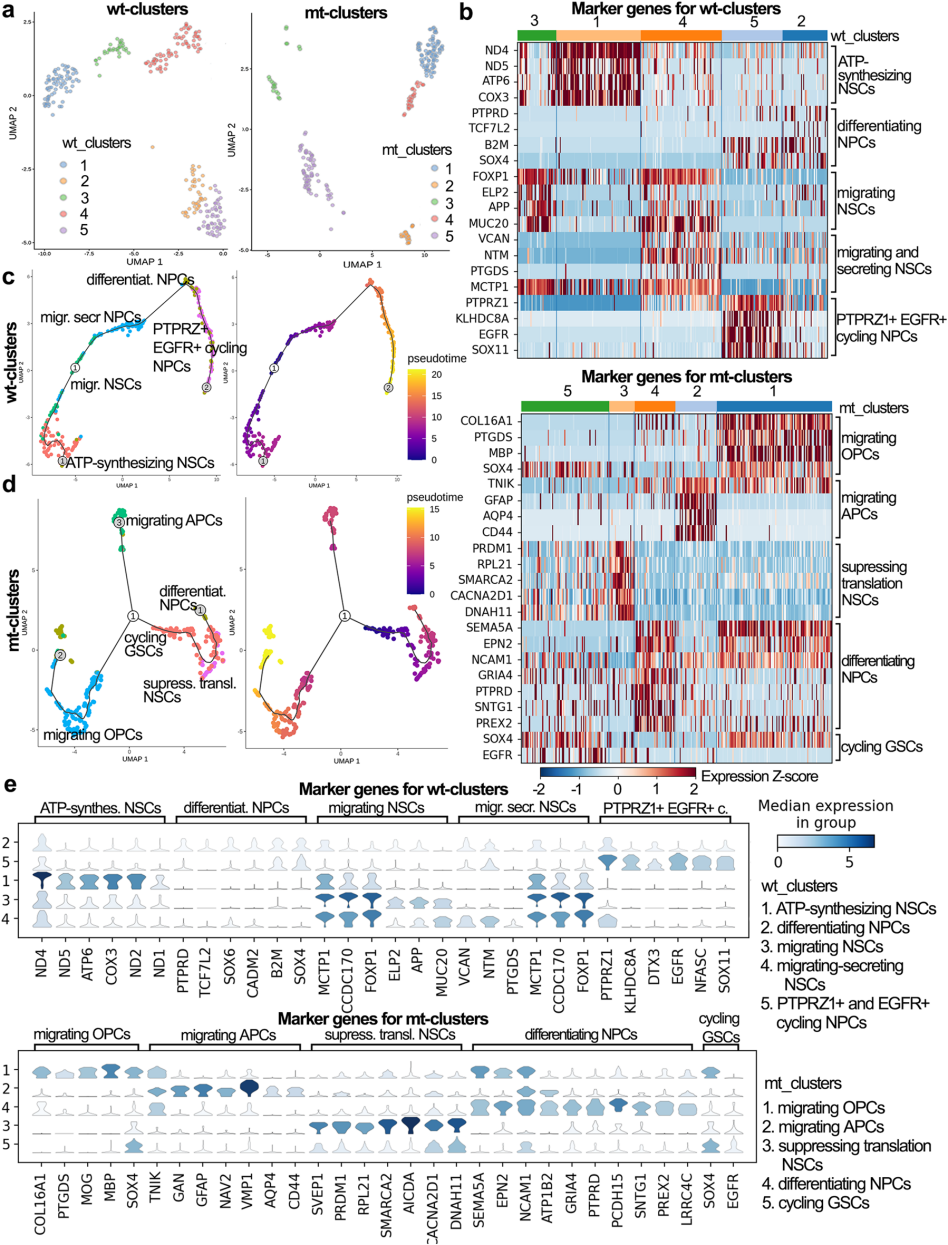
The difference between mutant and wild-type GSC clusters. (**A**) UMAP plot colored by clusters for wild-type (left) and mutant (right) samples. (**B**) Heatmaps highlighting expression of cluster-specific genes grouped by cell types (side description) and by UMAP clusters (upper color bar) for wild-type (superior) and mutant (inferior) samples. (**C**) Seurat clusters identified for wt-samples ordered in accordance with the RNA velocity (the first derivative of newly transcribed RNA and mature RNA) – a differentiation pseudotime by Monocle3. Colored by pseudotime—the shortest distance between a cell and the start of the trajectory and by Seurat clusters. (**D**) Seurat clusters identified for mt-samples ordered in accordance with the RNA velocity (the first derivative of newly transcribed RNA and mature RNA) – a differentiation pseudotime by Monocle3. Colored by pseudotime—the shortest distance between a cell and the start of the trajectory (right) and by Seurat clusters (left). (**E**) Stack violin plots displaying expression of cluster-specific genes annotated by cell types (upper description) and by UMAP clusters (side bar) for wild-type (superior) and mutant (inferior) samples. Color displays the level of gene expression.

Our next step was to separately analyze wild-type and mutant glioma cells. Wt-cells (283 cells) were subdivided using UMAP on five clusters (Fig. 4A): “ATP-synthesizing NSCs”, “differentiating NPCs”, “migrating NSCs”, “secreting and migrating NSCs”, “PTPRZ1 + and EGFR + cycling NPCs” (Fig. 4B, E). Signature genes of “ATP-synthesizing NSCs” as well as “PTPRZ1 + and EGFR + cycling NPCs” cells repeat previously described cluster 2 and cluster 4. The remained clusters were recognized as neural stem cells (NSCs) or neural progenitors (NPCs), as they upregulated neuronal signatures as well as genes regulating neurogenesis.

This way “differentiating NPCs” were considered as the most mature among NSC-like clusters, because they had neural genes (*PTPRD*, *CADM2*, *KIF5A*, etc.) in combination with regulators of neuronal differentiation – *TCF7L2*, *B2M* and *SOX6* maintaining stem state, as well as *SOX4* committing adult NSCs (Fig. 4B, E, Fig. S11A, Fig. S12A). “Migrating NSCs” were less differentiated cells, because they expressed more stem cell markers (*CCDC170*, *FOXP1*, *CBFB*), less neuronal genes (e.g. *MCTP1*, *APP*, *DST*, *SPG11*), and migration genes (*KIF14*, *MUC20*, *PHIP*) (Fig. 4B, E, Fig. S11A, Fig. S12A). “Secreting and migrating NSCs” regulated vesicle trafficking by *SAR1A*, *SPG11*, *SYT14* and migration genes – *VCAN*, *NTM* (Fig. S11A, Fig. S12A). “Differentiating NPCs”, “secreting and migrating NSCs”, and “migrating NSCs” represented parts of the “quiescent GSCs”.

Consistently, mt-GSCs also formed 5 clusters: “migrating OPCs”, “migrating APCs”, “suppressing translation NSCs”, “differentiating NPCs”, and “cycling GSCs” (Fig. 4A). Similarly, “migrating OPCs” and “migrating APCs” were described above (cluster 0 and cluster 5) (Fig. 3A).

Cells representing “suppressing translation NSCs” were defined by the genes associated with transcription and translation control (Fig. 4B, E). Specifically, *PRMD1* represses beta-interferon gene expression, *SMARCA2* belongs to the chromatin remodeling complex, *AICDA* participates in DNA demethylation, *ZC3HAV1* is a potent suppressor of general translation, *RPL21* is a component of the large ribosomal subunit. *CACNA2D1* and *DNAH11* are expressed in NSCs during differentiation to neurons^31,32^ (Fig. 4B, E, Fig. S11B, Fig. S12B). “Differentiating NPCs” from mt-samples were characterized by neuronal markers (*SEMA5A*, *NCAM1*, *GRIA4*, *PTPRD*, *SNTG1*, and *LRRC4C*), and a number of stem markers, e.g. *EPN2* activating Notch signaling^33^, *ATP1B2* distinguishing glioblastoma stem-like cells^34^, *PREX2* inhibiting tumor suppressor PTEN^35^ (Fig. 4B, E). “Cycling GSCs” from mt-samples upregulated *EGFR* and *SOX4* (Fig. 4B, E, Fig. S11B, Fig. S12B).

Analysis of wt-subpopulations identified “migrating NSCs” as a root population, which was closely connected to “migrating secreting NPCs”, and further differentiated into “PTPRZ + EGFR + cycling NPCs” through intermediate differentiating NPCs subpopulations (Fig. 4C). Meanwhile, mt-GSCs displayed three branches of differentiation – “migrating OPCs”, “migrating APCs”, and “differentiating NPCs” (Fig. 4D). Although, “cycling GSCs” population was the closest to the root of origin, the top hierarchical population was not identified for mt-GSCs (Fig. 4D).

### GSC markers due to mutations, malignancy, and grade

**Figure 5:**
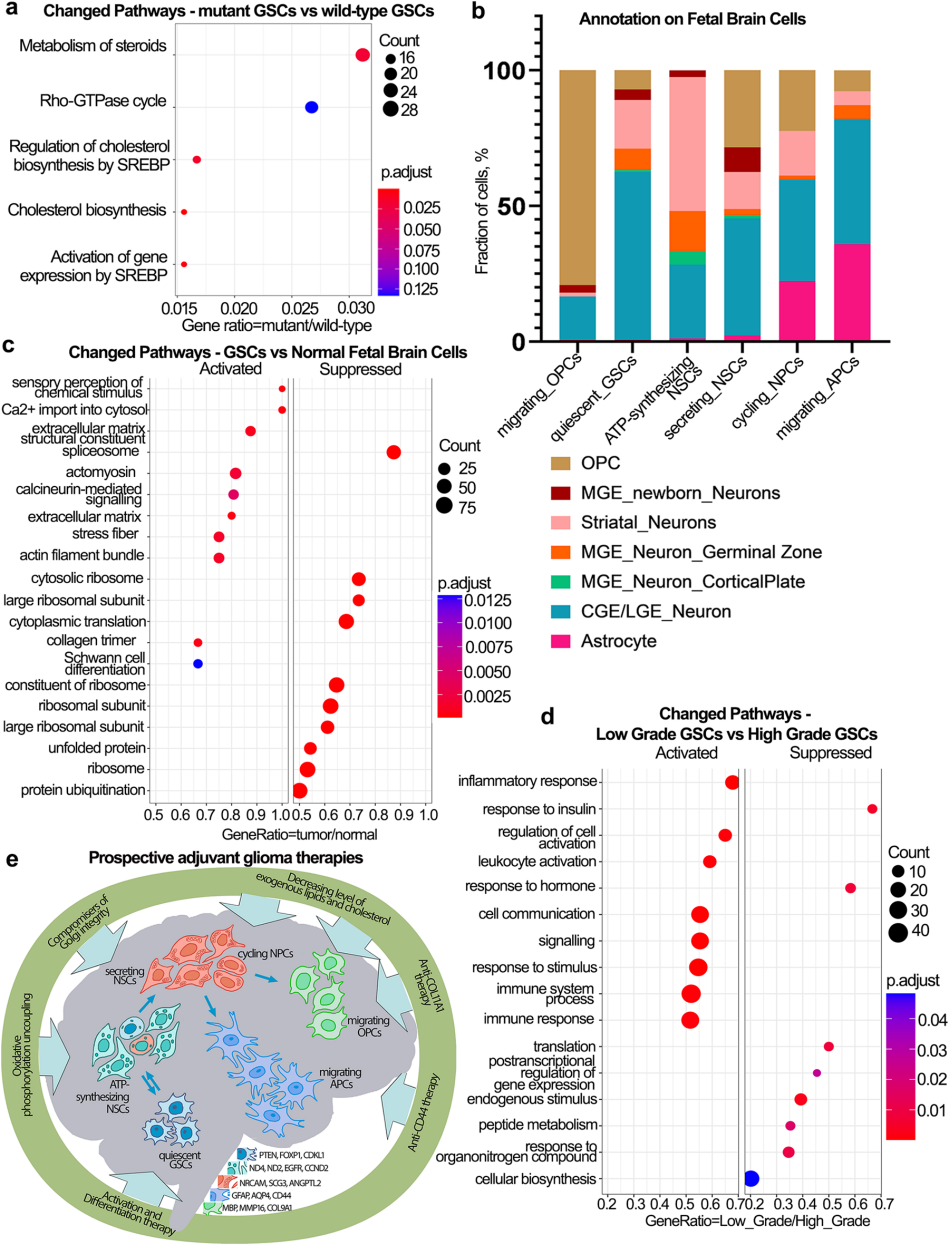
The possible molecular targets of GSC populations. (**A**) Dotplot enrichment map of differentially expressed genes of mt-GSCs compared to wt-GSCs. The size of sphere reflected the number of significantly changed genes, the sphere color displayed the significance of the changes occurred in the corresponding metabolic pathway. (**B**) Barplot showing the composition of UMAP GSC clusters annotated using normal fetal brain single cell data. Each bar shows the proportion of cells (%) assigned as normal fetal brain cells annotated in color bar on the right side. (**C**) Dotplot enrichment map of differentially expressed genes comparing UMAP GSC clusters to normal fetal brain cell clusters which were presented in our dataset. The size of sphere reflected the number of significantly changed genes, the sphere color displayed the significance of the changes occurred in the corresponding metabolic pathway. (**D**) Dotplot enrichment map of differentially expressed genes comparing low-grade samples to high-grade samples within our dataset irrespective of the mutations. The size of sphere reflected the number of significantly changed genes, the sphere color displayed the significance of the changes occurred in the corresponding metabolic pathway. (**E**) Possible adjuvant therapeutic approaches for each GSC populations according to their molecular profile. Molecular markers of the GSC populations are listed in the bar in the lower part. Small arrows annotate the differentiation path. Big arrows annotate the suggested therapeutic approaches.

Gene ontology analysis revealed that mt-GSCs downregulated metabolism of steroids and cholesterol biosynthesis (Fig. 5A), specifically, the enzymes implicated in the cholesterol production, e.g. *ACACA*, *ACACB*, *HMGCR*, etc. (Fig. S13A). IDH1 R132H intensively consumes NADPH^36^, that is involved in lipogenesis, so low NADPH could disrupt the Fatty acid synthase activity. Additionally, mt-GSCs displayed enhanced ERBB and Wnt signaling pathways combined with upregulation of actin cytoskeleton, focal adhesion pathway, expression of cell adhesion molecules (Fig. S13B-S13E).

We compared GSCs to normal fetal brain cells from Nowakowski^37^. To understand which cell types forming normal fetal brain represent the most suitable control, we annotated our GSC clusters on Nowakowski clusters. Interestingly, most of GSC clusters contained CGE/LGE (caudal/lateral ganglionic eminence) neurons posed to migration under normal condition^38^. Clusters “migrating OPCs” and “migrating APCs” had big proportion of OPCs and astrocytes, respectively (Fig. 5B). Differential expression analysis showed, that GSCs activated pathways connected to cell migration and endoplasmic reticulum stress (“extracellular matrix”, “actomyosin”, “Ca2 + import”, etc.) (Fig. 5C – Activated). Meanwhile, compared to normal cells, GSCs suppressed translation and protein degradation (Fig. 5C – Suppressed).

Additionally, we compared low-grade glioma samples (G19 and Rel1) to high-grade ones (G9, G16, G18, G20) irrespectively of the mutational profile and cluster annotation. Low-grade gliomas showed activated immune response and inflammation, while they displayed downregulated response to hormones, translation and overall biosynthesis (Fig. 5D). Thus, high-grade gliomas characterized by the lack immune inflammation and intensive biosynthesis.

After exploring the GSCs markers, we suggest the different GSC subpopulations could be targeted by the diverse therapeutic approaches (Fig. 5E). For more differentiated GSCs, such as “migrating APCs” and “migrating OPCs”, we propose antigen specific therapeutic strategies, CD44 and collagen XI respectively. Decreasing the level of exogeneous lipids and cholesterol would be a proper recommendation to stop the GSCs with the high proliferation index (“cycling NPCs”, “ATP-synthesizing NSCs”) and IDH1-mutant GSCs. The least defined populations – “secreting NSCs” and “quiescent GSCs” – can be targeted with unspecific differentiation therapy.

### Mutant glioma cells display CD44^high^/Nestin^low^ phenotype and high migration ability

Although, in vitro cell cultures carry inherent disadvantages, so far, patient-derived primary cultures are commonly applied for GSC research^39^. Heterogeneous primary glioma preserve phenotypical features and have similar stem cell properties in vitro^40^. Using primary glioma cultures, we inquired the main observed transcriptomic effects, namely, stem-proliferative phenotype for wt-GSCs and differentiated-migrating phenotype for mt-GSCs.

**Figure 6:**
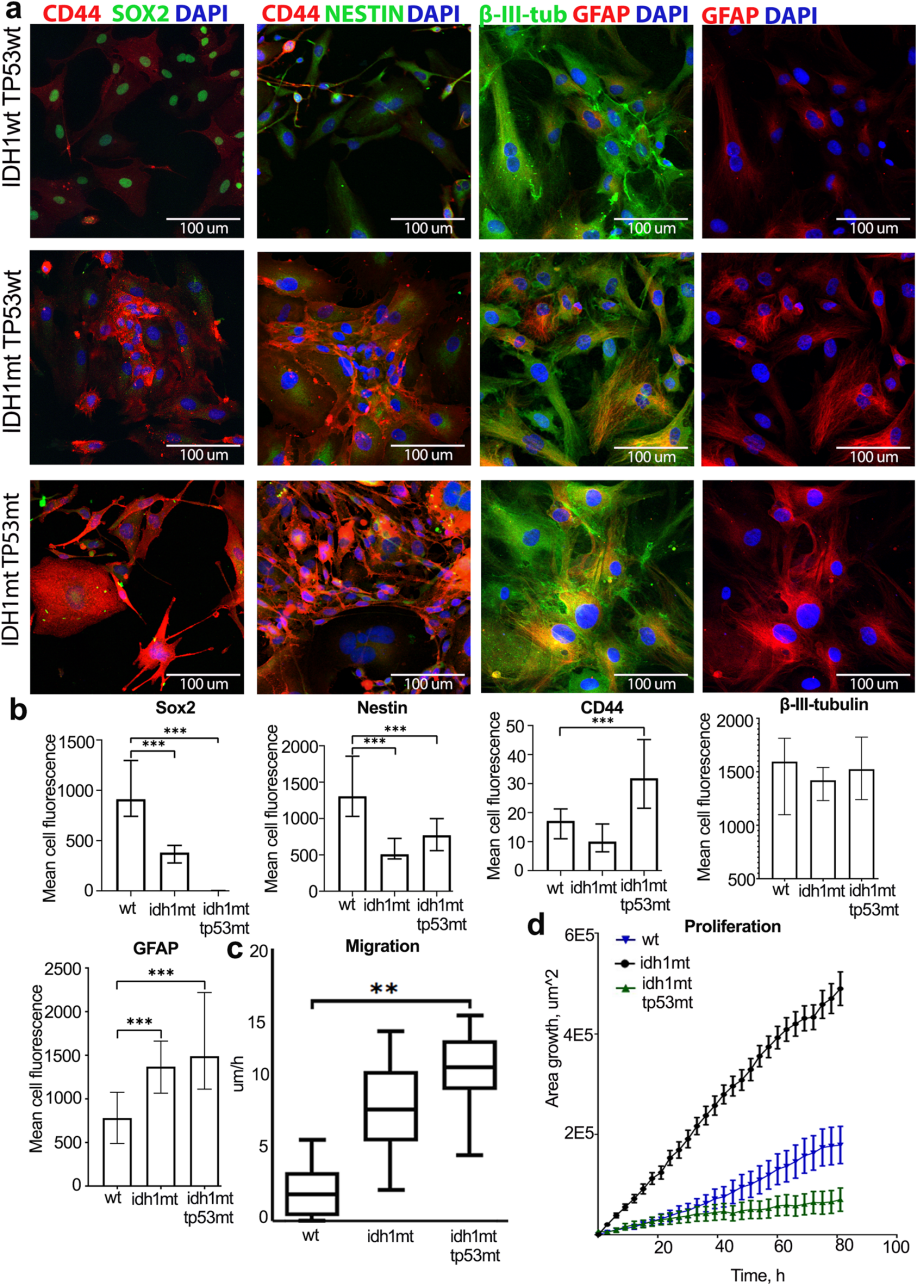
Stemness, migration and proliferation of wild-type and mutant cell cultures. (**A**) Staining of glioma primary cells (IDH1wt_TP53wt cells – superior pictures, IDH1mt_TP53wt – middle pictures, IDH1mt_TP53mt – inferior pictures) for stem (Sox2 and Nestin), migration (CD44) markers, and differentiation markers (GFAP and β-III-tubulin). CD44 and GFAP displayed in red, Sox2, Nestin, and β-III-tubulin in green, DAPI in blue. (**B**) Sox2, CD44, Nestin, GFAP and β-III-tubulin expression by primary glioma stem cells. Expression was counted as mean fluorescence intensity for IDH1wt and TP53wt (wt) cells, IDH1mt-cells, and IDH1mt-TP53mt cells. Data presented as median ± Q1/Q3, statistical significance was set to *p* < 0.05. (**C**) Migration velocity measured in um per hour of wt-cells, IDH1mt-cells, and IDH1mt-TP53mt cells. The data presented as mean ± SD for n = 4 replicates, statistical significance was set to *p* < 0.05. (**D**) Proliferation speed measured in area of growth (um2) per hour of wt-cells, IDH1mt-cells, and IDH1mt-TP53mt cells. The data presented as mean ± SD for n = 4 replicates.

IDH1-mutant (IDH1mt) and TP53-mutant (TP53mt) glioma cells highly expressed CD44, but completely missed Sox2 and downregulated Nestin. Solely IDH1mt cells expressed downregulated all measured stem cell and migration markers, with a trace staining. On the contrary, wt-cultures expressed high Sox2 and Nestin with relatively low CD44 (Fig. 6A, Fig. 6B, Fig. S14). Additionally, the proportion of Sox2 + cells in wt-cultures was around 50%, and almost all cells displayed intensive intra nucleus staining, while IDH1mt glioma cells revealed sparse Sox2 staining localized in both cell nucleus and cytoplasm (Fig. 6A, Fig. S14). Moreover, we assessed the expression of neural cell differentiation markers – GFAP (glial lineage) and β-III-tubulin (neuronal lineage) and uncovered the highest commitment of IDH1mt and IDH1mt-TP53mt cells to glial differentiation. There was also a tendency in IDH1wt-TP53wt cells to express β-III-tubulin higher, although it was not significant (Fig. 6A, Fig. 6B).

Next, we investigated, whether mutant glioma cells expressing high CD44 were prone to migration. IDH1mt and TP53mt cells migrated almost three times faster than wt-cells (Fig. 6C). At the same time wt-cells proliferated almost 5 times faster than IDH1mt and IDH1mt/TP53mt cells (Fig. 6D). Additionally, there was no significant difference in the proliferation speed between IDH1mt and IDH1mt/TP53mt cells (Fig. 6D) emphasizing IDH1 R132H as a main restraint of their cell cycle.

## Discussion

The current study aimed at identifying molecular targets driving survival of the *IDH1* and *TP53*-mutant GSCs. Mt-GSCs were characterized by increased migration activity, APC or OPC-like phenotype. Additionally, mt-GSCs revealed increased production of collagens which construct GSC niche and make tumor interstitium stiffer precluding drug penetrance^41–43^. High expression of collagens is a feature of quiescent GSC with mesenchymal profile^44^ and can be attributed to skewed function of *TP53* GOFs^45,46^. Additionally, mt-GSCs downregulated the genes responsible for the cholesterol production and fatty acid metabolism, which make mutant cells dependent on exogenous lipids^47^. *IDH1* R132H consumes NADPH and leads to low lipid metabolism and disruption of lipid rafts, that can potentiate loss of cell adhesion and subsequently higher migration^48,49^. Summarizing, we suggest mt-GSC vulnerability points: (i) collagen synthesis, (ii) CD44 expression, and (iii) cholesterol/lipid metabolism. Wt-GSCs were (i) ATP-synthesizing, (ii) proliferating, (iii) neuronal-like.

Differentiated classes of GSCs can be reached by antigen-specific therapy, anti-CD44 antibody for “migrating APC” and anti-collagen XI – for “migrating OPCs” (Fig. 5E). High level of lipid biosynthesis is essential for rapidly cycling cells^50^, however the use of lipid biosynthesis inhibitors can be ineffective, due to consumption of exogeneous lipids from the blood^51^. Therefore, the glioma patients could use statins decreasing LDL level or follow the diet with decreased level of lipids and cholesterol. Additionally, the rapidly proliferating cells, relying on mitochondria for efficient ATP production, can be targeted by the oxidative phosphorylation uncouplers^52^. “Secreting NSCs” upregulated the activity of Golgi apparatus, which integrity can be compromised^53^. “Quiescent GSCs” are the most difficult to target, but can be forced to differentiation by retinoic acid^54^.

The new GSC paradigm claims the absence of GSC populations, but, rather, a continuous transition of glioma cell states characterized in terms “less or more differentiated progenitor-like cells”. Normal stem cell differentiation can be also considered as a continuous change of marker gene expression. The main difference between normal and glioma stem cells is the reversibility of the differentiation process. Therefore, stem cell marker expression is still a hallmark of cell state potency either normal or tumor. Thus, we profiled Sox2 + glioma cells to capture rare GSC populations, that can be vanished in big studies observing all cells within a tumor.

The GSCs in our dataset displayed a certain hierarchy, which can be modelled in accordance with the adult NSC differentiation model^28^. Accordingly, activated NSCs divide asymmetrically into a dormant cell and a transient amplifying progenitor cell. In our dataset, “ATP-synthesizing NSCs” divide into “quiescent GSCs” and “cycling/secreting NSCs/NPCs”, that upon a number of symmetric divisions differentiate into invasive “migrating OPCs/APCs”. The latter subpopulations might migrate within brain tissue and form new colonies. As the main difference of the normal and tumor differentiation processes is the reversibility of the latter^55^, “migrating OPCs/APCs” can dedifferentiate back to active/quiescent GSCs, thus forming a new self-sufficient tumor.

The staining for cell differentiation factors alludes that the cell of origin for wt- and mt-tumors could be different in the sense that IDH1mt-TP53mt cells were poised to glial branch of differentiation (GFAP expression), while IDH1wt-TP53wt could be prone to neural branch (β-III-tubulin expression).

The limitations of the present study can be outlined by the absence of the appropriate healthy control group, incorporation of different point mutations for *TP53* gene (R248Q, R273C – as outlined in the Table 1). One of the samples, included in the scRNAseq experiment, was Rel1 with solely *IDH1* R132H mutation. This sample was too small to provide the statistically significant difference to infer the difference between *IDH1*- and *TP53*-double mutant GSCs and *IDH1*-single mutant cells. Since, *IDH1*-single mutant sample was clustered with the double mutant samples, we decided to incorporate Rel1 in the mutant cohort. The reason for exploring the double mutant scenario is observed high frequency of *IDH1* and *TP53* mutations in combinations (Fig. 1A). Additionally, IDH1mt cell cultures appeared to exhibit cytoplasmic Sox2 staining, that can be due to its proteolytic degradation as a result of cell differentiation^56^, a pattern characterizing a particular antibody used^57^, expression localization in particular cell types – trophoectoderm cells^58^. In our case, the most probable explanation would be the acetylation of Sox2, which take place in response to differentiation signals and leads to Sox2 nuclear export^59^.

In conclusion, our study adds new insights in the complexity of *IDH1-* and *TP53*-mutant and wild-type GSCs highlighting several prospective target mechanisms that can be reverted against GSCs: (i) collagen synthesis, (ii) lipid/cholesterol synthesis, (iii) antioncogene expression. Wild-type GSCs did not display unified molecular marker and could require the establishing differentiation therapy protocol. Meanwhile, *IDH1*- and *TP53*-mutant gliomas possessing more differentiated GSC subpopulations, diversified on oligodendrocyte- and astroglia-like phenotypes, that could be targeted by their common property of migration/invasion, using adhesion molecules, such as CD44.

## Materials and methods

### Patient tumor samples

All tumor samples were de-identified and provided by Far Eastern Federal University (FEFU) Medical Center. The patient sample use was approved by the FEFU Ethics Committee according to Ref. #5/12-19-2017, all the experiments were set under the principles of WMA Declaration of Helsinki. All patients willing to participate in the study signed the informed consent. The clinical diagnosis for de-identified patient samples was provided by FEFU Medical Center based on immunohistochemical analysis. The received tumors were collected in the sterile cold PBS and divided into pieces intended for transcriptomics, genotyping and cell culture establishment. Tissues for genetic test and transcriptomics were snap-frozen in the liquid nitrogen, the piece imposed for cell culture was dissociated under sterile conditions within 2 h after receiving the tissue. Table 1 contains the information about patients.

### Glioma primary cell cultures establishment

Glioma primary cell cultures were set up by tumor tissue mincing and enzymatic dissociation in the dissection buffer with 1 mg/ml collagenase/dispase (Roche, USA) and 1 mg/ml DNAse I (Roche) for 30 min in the incubator (37 °C, 5% CO_2_) with concurrent shaking every 5 min. Then the pieces were gently piped up and after settling down the supernatant with dissociated cells was transferred in the cold wash buffer (3% FBS (Gibco, USA) in PBS (–Ca^2+^, –Mg^2+^) (Gibco)). The procedure was repeated until the pieces were completely dissociated and pulled in the wash buffer, then centrifuged at 200 g for 5 min at 4 °C and transferred in the Ham's F-12 Nutrient Mix with GlutaMAX (Gibco) containing 20% FBS. The next day dead cells were washed out with PBS while the adhered ones were further cultivated in DMEM/F12 (Gibco) containing 10% FBS. Glioma cell cultures were considered stable after at least three passages and reaching total cell count approximately 5*10^6^–10^7^ cells, then frozen in liquid nitrogen for cryopreservation.

### Cell migration and proliferation analysis

Cell migration assay was performed using high-content imaging system Cell-iQ MLF (CM Technologies, Finland). BT29 (IDH1 R132H/wt TP53 R248Q/R248Q), BT32 (IDH1 R132H/wt TP53 wt/wt), and BT40 (IDH1 wt/wt TP53 wt/wt) cell cultures were plated in tissue-culture treated flat-bottom 24-well plates (Corning, USA) at a density of 15,000 cells/cm2. Cells were maintained in 1 ml of DMEM/F12 supplied with 10% FBS. After 90 h of cultivation with live-cell time-laps monitoring, cells were analyzed for migration velocity applying the Cell-iQ Analyzer software. Each cell culture was analyzed in 4-well replicates, and at least 9 view-fields in each well were recorded using time-lapse mode. Cell motility was analyzed to reach more than 450 individual cell tracks for each glioma culture. A total of 640 cells for BT29, 476 cells for BT39 and 632 cells for BT40 participated in the analysis. Cell migration data are presented as Tukey plots of average velocity over 90 h.

The same primary glioma cell cultures were used for the proliferation analysis which was performed on the same imaging system described for migration. The proliferation rate was measured during 4 days as the average number of cells in three wells at each moment of shooting. The cell cultures were plated in tissue-culture treated 24-well plates at a density of 13,000 cells/cm^2^. All cell cultures were plated at three replicates.

### Immunocytochemistry

We used sandwich method for immunohistochemistry (ICH) analysis. For ICH cells were seeded in the 8 well chamber slide (Ibidi, Germany) with density – 10 thousand cells per well in 200 ul complete medium. Two days after seeding, the cells were fixed in 10% paraformaldehyde (Sigma) in PBS (pH 7.4) (Gibco) for 10 min at room temperature (RT), washed three times in PBS with 0.05% Tween-20 (Helicon, Russia) for 5 min at RT, permeabilized the membranes by incubating in PBS with 0.5% Triton X100 (Helicon) for 10 min at RT, again triply washed in PBS with 0.05% Tween-20 for 5 min at RT, and finally incubated in 3% non-fat dry milk (Carl Roth, Germany) at + 4 °C overnight. After incubation with primary antibodies for 2 h at RT, cells were washed three times with PBS and incubated with secondary antibodies for 1 h at RT in the dark. After washing, cells were stained with 1 ug/ml DAPI (Invitrogen) for 5 min at RT in the dark, washed three times with PBS, and mounted in Mowiol (Sigma) with N-propyl gallate (Sigma). Anti-Sox2 rabbit polyclonal (ab97959, Abcam, UK) at the concentration of 1 ug/ml, anti-CD44 mouse monoclonal (ma5-13,890, Invitrogen) at the concentration of 2 ug/ml, anti-Nestin rabbit polyclonal (pa5-79,729, Invitrogen) at the concentration of 1 ug/ml, anti-β-Tubulin III polyclonal rabbit (T2200, Sigma) at 5 ug/ml, anti-GFAP monoclonal mouse at the concentration of 2.5 ug/ml (GA5, Invitrogen) were used as primary antibodies. Secondary antibodies included Alexa Fluor 488 goat anti-rabbit IgG (H + L) (a11034, Invitrogen) and Alexa Fluor 546 goat anti-mouse IgG (H + L) (a11003, Invitrogen), both used in dilution 1:500. The samples were analyzed using FluoView FV1200MPE-FV12M-5XX-3XX laser scanning microscope (Olympus, Japan). The pictures for samples BT40 (wild-type), BT32 (IDH1 R132H), BT29 (IDH1 R132H and TP53 R248Q) were analyzed by measuring mean cell fluorescence for n = 50 cells for each patient cell line using ImageJ software. To affirm the staining results, we included pictures for additional cell lines – BT35, G1, BT39, U87MG with introduced IDH1 R132H/wt and TP53 R248Q/R248Q. The information about additional cell lines can be found in Table S6. The standard U87MG with introduced mutations was included due to very poor survival of double mutant (IDH1 R132H and TP53 missense mutation) primary cell lines and, consequently, our inability to prove the concept other way.

### Tumor sample genotyping

Genomic DNA was extracted from snap frozen tumor tissue samples with GeneJET Genomic DNA Purification Kit (Thermo Fisher Scientific, USA) according to the protocol provided by the manufacturer. The gene fragments containing regions of interest for IDH1, IDH2, TP53, and BRAF were amplified using DreamTaq Green PCR Master Mix (2X) (Thermo Scientific). List of primers used for PCR is described in Table S1. Then the PCR mix was purified using Cleanup Mini kit (Evrogen, Russia) and the DNA concentration in every tube was measured using Nanodrop Spectrophotometer (Implen). Next 10 ng of purified DNA was applied to Sanger Sequencing by BigDye Terminator v3.1 Cycle Sequencing Kit (Applied Biosystems, USA).The reaction protocol was 96 °C – 1 min; 25 cycles: 96 °C – 10 s, 55 °C – 10 s, 60 °C – 4 min; 4 °C – hold. Further, the sequencing reaction was purified using the BigDye XTerminatorTM Purification Kit (Applied Biosystems) and sequenced on the 3500 Genetic Analyzer (Applied Biosystems). Chromatogram analysis was performed with SnapGene Viewer (GSL Biotech, USA).

### Tissue lysis and nuclei staining

Frozen tumor tissue of approximate size 0.2–0.5 cm3 was placed in 5 ml of the cold tissue lysis buffer (RNase-Free Water (IDT, USA), 5 M NaCl (Sigma, USA), 1 M MgCl2 (Sigma), 1 M Tris buffer (pH 8.0) (Sigma), 10% NP40 (Sigma), 1uM DTT (Invitrogen), 1 × Complete protease inhibitor (Roche), 0.4 U/ul RNAseIn (Takara Bio, Japan), 0.2 U/ul SuperaseIn (Thermo Fisher Scientific)) in 50 ml tube (Eppendorf, Germany) and incubated on ice for 15 min with gentle swirling every 2–3 min. Then the tissue was triturated, filtered through 40um cell strainer (Corning) and centrifuged at 1000 g for 5 min at 4 °C. The sedimented nuclei were resuspended in 1 ml cold staining buffer (RNAse-free PBS pH 7.4 (Invitrogen), 1% BSA (Sigma), RNAseIn, 1 M MgCl2, 1 mM DTT) and stained with anti-Sox2 polyclonal antibody (Invitrogen, #48-1400) in concentration 2 ug/ul or respective IgG isotype control (BD, Clone Poly1281 (RUO), #550,875) in concentration 1 ug/ul. The staining with first antibodies was performed at 4 °C while rotating for 45 min. Alexa Fluor 488 Goat anti-Rabbit IgG antibody (Invitrogen, #A-11008) in concentration 4 ug/ul was used as secondary. The staining was done in the dark at 4 °C for 30 min while rotating. Nuclei were visualized with 7-Aminoactinomycin (7-AAD) (Sigma) staining in the concentration 6.7 ug/ul. Thereafter, Sox2 positive nuclei were sorted (1 nucleus per well) at FACS Aria I or III at 4 °C in skirted 96-well PCR plates (Thermo Fisher Scientific) containing 2 ul cell lysis buffer (2U/ul RNAseIn in 0,2% (vol/vol) Triton X-100 (Sigma)). After sorting plates were immediately sealed and placed on dry ice. Plates were either used directly for cell lysis or stored at − 80 °C for no more than a month.

### Single nuclei smart-seq2

Smart-seq2 protocol for single nuclei sequencing was performed as described in^60^ with several modifications^61^. In brief, individual nuclei were lysed, RNA lysate was reverse transcribed with 100U of Superscript III (Takara) with the addition of 10U of RNaseIn, 5 mM DTT, 1 M betaine (Sigma), and 6 mM MgCl2 (Invitrogen). Reverse transcription reaction was paused after 90 min at 42 °C for adding TSO oligos at the final concentration of 0.5 uM and continued for additional 12 min. The cDNA was amplified using KAPA HotStart HIFI 2 × ReadyMix (Kapa Biosystems) and 10 uM ISPCR primer for 24 cycles (98 °C – 3 min; 10 cycles: 98 °C – 20 s, 60 °C – 1 min, 72 °C – 6 min; 7 cycles: 98 °C – 20 s, 64 °C – 1 min, 72 °C – 6 min; 7 cycles: 98 °C – 20 s, 67 °C – 1 min, 72 °C – 6 min; 72 °C – 10 min; 4 °C – hold). Amplicons were purified by means of Ampure XP beads (Beckman Coulter, USA) at a sample:bead ratio of 1:1, their concentration was assessed using Qubit dsDNA HS Assay Kit (Invitrogen), while the amplicon quality was estimated by cDNA length on high-sensitivity DNA chip (Agilent) on Agilent 2100 Bioanalyzer. Oligo-dT, TSO and IS PCR primers (sequences are listed in Table S2) were ordered from Exicon (http://www.exiqon.com/). All primer sequences are listed in the Table S1.

### Library preparation for RNA-sequencing

The library preparation was done using Nextera XT DNA Library Preparation Kit (Illumina, USA) according to the manufacturer instructions. The Illumina Nextera Adapters (sequences provided on ngs.biodiv.tw/NGSCore/wp-content/uploads/Documents/illumina-adapter-sequences-1000000002694–08.pdf) were synthetized and HPLC purified in Biomers (http://www.biomers.net/). 0.2 ng of input cDNA was tagmented and amplified with Illumina Indexes (i7 and i5) for 12 cycles. After purification with SPRI beads (Beckman Coulter) at a sample:bead ratio of 0.6:1, each library quality was assessed using Agilent HS DNA assay and the concentration was measured by Qubit DNA HS Kit. The molarity of each library was brought to 2 nM and then pooled together. After denaturation and dilution to 1.6–1.7 pM, the libraries were sequenced on an Illumina NextSeq 500. Single-end 75 bp sequencing was performed on the Illumina HiSeq 2500.

### Single cell RNAseq data analysis

Raw fastq files were subjected to quality control with FastQC (version 0.11.9) and trimming Nextera sequencing adapters with Trim Galore (parameter “–nextera”). Then trimmed files were mapped to GRCh38.p12 human reference genome (accessible from https://www.ncbi.nlm.nih.gov/assembly/GCF_000001405.38) using STAR-2.6.0c with the parameters ‘–outFilterMatchNmin 17 –seedSearchStartLmax 30 –outFilterScoreMinOverLread 0 –outFilterMatchNminOverLread 0’. In order to generate counts for aligned reads we used STAR ‘–quantMode GeneCounts’ which produced *ReadsPerGene.out.tab files for each fastq file. Column 2 containing counts for unstranded RNA-seq from *ReadsPerGene.out.tab files were merged to generate count matrix. STAR is free open source software that can be obtained by General Public License (GPLv3 license). The source for download is http://code.google.com/p/rna-star/ or https://github.com/alexdobin/STAR.

Genes expressed in neither one cell were removed from the analysis. Blood cells were excluded from the analysis by expressing hematopoietic markers (all hematopoietic cell—CD45 (*PTPRC*); naïve CD4 + T cells – *IL7R*, *CCR7*; memory CD4 + cells – *S100A4*; monocytes – *CD14*, *LYZ*, *FCGR3A*, *MS4A7*; B-cells – *MS4A1* (CD20); CD8 + T cells – *CD8A*; NK-cells – *GNLY* (NKG5), *NKG7*; dendritic cells – *FCER1A*, *GST3*; platelet – *PPBP*; stromal cells – *CD34*; endothelial cells – *PECAM1* (CD31)).

The rest cells were analyzed for the presence of big rearrangements (deletions or amplifications) referred to as copy number variations (CNVs) using CONICSmat^62^. The minimum of the counts was obtained for every heterozygous single nucleotide polymorphism, summed for every chromosome, and divided by the total counts for every chromosome to obtain an allelic imbalance ratio^63^.

Cell cycle classification was performed by means of Cyclone^25^ assigning cells to G1, S, G2/M phases on the basis of the cell-cycle genes. CellCycleScoring() of the Seurat on R basis was used to confirm the cell-cycle distribution through the canonical phases. ccAF^24^ on python basis was used to determine cells presented in G0 phase.

For the population analysis we used SingleCellExperiment^64^, Seurat^65^, and Scanpy^66^. The populations were divided using Leiden method and embedded by the UMAP. All cells were assigned to functional classes described on the basis common function joining the biggest number of genes in each cluster. Genes characterizing all the populations were revealed by differential expression analysis employing Limma^67^ and DEsingle^68^. Gene ontology analysis and altered pathway visualization was done via KEGG^69–71^ and Reactome PA^72^.

The annotation of the single cell clusters was further verified by means of scMatch^26^ identifying a cell type of a cluster by matching gene expression profiles to the large reference datasets.

The differentiation trajectories were created by Monocle 3^73^ by fitting the models of gene expression as a function of pseudotime.

To annotate GSC clusters on Nowakowski dataset^37^, we first ranked the genes in each cell of normal fetal brain dataset and constructed the curve of marker genes (genes with the highest expression). Then employing AUcell package^74^ in R, we computed the area under the curve (AUC) for all marker genes for each cell and quantified the most enriched gene set among marker genes. To assign our GSCs to have fetal brain cell identity, we took top AUC sets of genes as markers for labeling GSCs. The procedure and mathematics behind is described in the Bioconductor source (http://bioconductor.org/books/3.13/OSCA.basic/cell-type-annotation.html). Nowakowski cortex data were obtained as SingleCellExperiment object by running NowakowskiCortexData() with prerequisite of scRNAseq library. All normal brain clusters containing less than 5 glioma cells were omitted to get the general picture. To align IDH1wt dataset GSM3828672 and IDH1mt dataset GSE89567, from which we chose double IDH1mt-TP53mt samples (Table S5), with the identified Seurat clusters, we implemented the same approach but vice versa. Additionally, we excluded Sox2-negative cells (the expression in row counts was equal to 0) from the data analysis to focus only on glioma stem cells.

The differential expression analysis of normal vs tumor cells, as well as low-grade vs high-grade cells was performed by aggregating the raw counts values for GSC clusters or glioma samples respectively. Using the pseudobulk analysis (https://hbctraining.github.io/scRNA-seq/lessons/pseudobulk_DESeq2_scrnaseq.html), we performed differential expression by means of “limma” package in R environment. The differentially expressed genes having p.adjust < 0.05 were further analyzed by gene set enrichment analysis – gseGO() and plotted with the “DOSE” package.

The Cancer Genome Atlas (TCGA; https://www.cancer.gov/tcga) data were inquired from the R environment using libraries RTCGA.clinical and RTCGA.mutations. The patients were selected to have both mutations – double_mut (IDH1 missense and TP53 missense), IDH1_solely (IDH1mutant subtracting double_mut), TP53_solely (TP53 mutant subtracting double_mut), wild_type (all_patients subtracting IDH1_solely, TP53_solely, double_mutant).

For survival curves we quired cBioPortal^75,76^, where we chose CNS/Brain tab. For LGG patients we chose Brain Lower Grade Glioma (TCGA, Firehose Legacy) and (TCGA, PanCancer Atlas). After inquiring IDH1 and TP53 genes in the Gene Query, we ran the analysis. For the GBM analysis we set Glioblastoma (TCGA, Cell 2013), (TCGA, Nature 2008), Glioblastoma Multiforme (TCGA, Firehose Legacy), (TCGA, PanCancer Atlas).

### Statistics

For experiments performed on primary cell lines, statistics were calculated using GraphPad Prism v8.0.1 software. For immunocytochemical analysis data for n = 50 cells were acquired from 4 individual Ibidi chamber wells and analyzed for the expression of markers CD44, Sox2 and Nestin. The data normality distribution was enquired by means of Kolmogorov–Smirnov test. The statistical significance of the both marker expression was calculated using Kruskal–Wallis test. The critical level of significance was taken equal to 0.05. Results are presented as median ± quartile 1/3 (Q1/3). The indicators of migration and proliferation were analyzed for 9 central view-fields in 4 replicates for each cell line using automated cell recognition and applying one-way Anova. The data presented as mean ± standard deviation (SD).

## Ethical approval

The study was conducted according to the guidelines of the Declaration of Helsinki, and approved by Ethics Committee of the Far Eastern Federal University according to Ref. #5/12-19-2017.

## Supplementary Information

Supplementary Information.

## Data Availability

All data generated during this study are included in this published article and its supplementary files. Raw sequencing data and processed gene expression data were deposited at the Gene Expression Omnibus (GEO) under accession number GSE164624.

## Abbreviations

IDH1: Isocitrate dehydrogenase 1
GOF: Gain-of-function mutation
GSCs: Glioma stem cells
CSCs: Cancer stem cells
TCGA: The Cancer Genome Atlas
snRNAseq: Single nucleus RNA sequencing
NSC: Neural stem cell
NPC: Neural progenitor cell
OPC: Oligodendrocyte progenitor cell
APC: Astrocyte-precursor cells
mt-samples, mt-group, mt-GSCs, mt-cells: *IDH1*- and *TP53*-mutant samples
wt-samples, wt-group, wt-GSCs, wt-cells: *IDH1*- and *TP53* wild-type samples
IDH1mt: IDH1-mutant
TP53mt: TP53-mutant
IDH1wt: IDH1 wild-type
TP53wt: TP53 wild-type
ECM: Extracellular matrix

## References

[1] Louis DN (2016). The 2016 world health organization classification of tumors of the central nervous system: a summary. Acta Neuropathol..

[2] Shajani-Yi Z, de Abreu FB, Peterson JD, Tsongalis GJ (2018). Frequency of somatic TP53 mutations in combination with known pathogenic mutations in colon adenocarcinoma, non-small cell lung carcinoma, and gliomas as identified by next-generation sequencing. Neoplasia (New York, N.Y.).

[3] Morrison SJ, Kimble J (2006). Asymmetric and symmetric stem-cell divisions in development and cancer. Nature.

[4] Hanel W (2013). Two hot spot mutant p53 mouse models display differential gain of function in tumorigenesis. Cell Death Differ..

[5] Suvà ML, Tirosh I (2020). The glioma stem cell model in the era of single-cell genomics. Cancer Cell.

[6] Bhaduri A (2020). Outer radial glia-like cancer stem cells contribute to heterogeneity of glioblastoma. Cell Stem Cell.

[7] HassnMesrati M, Behrooz AB, Abuhamad YA, Syahir A (2020). Understanding glioblastoma biomarkers: knocking a mountain with a hammer. Cells.

[8] Sarlak G, Vincent B (2016). The roles of the stem cell-controlling Sox2 transcription factor: From neuroectoderm development to Alzheimer's disease?. Mol. Neurobiol..

[9] Episkopou V (2005). SOX2 functions in adult neural stem cells. Trends Neurosci..

[10] Takahashi K (2007). Induction of pluripotent stem cells from adult human fibroblasts by defined factors. Cell.

[11] Vasquez JC (2017). SOX2 immunity and tissue resident memory in children and young adults with glioma. J. Neurooncol..

[12] Saenz-Antoñanzas A (2021). CRISPR/Cas9 deletion of SOX2 regulatory region 2 (SRR2) decreases SOX2 malignant activity in glioblastoma. Cancers (Basel).

[13] Li P (2022). Selective single-cell expansion on a microfluidic chip for studying heterogeneity of glioma stem cells. Anal. Chem..

[14] Couturier CP (2020). Single-cell RNA-seq reveals that glioblastoma recapitulates a normal neurodevelopmental hierarchy. Nat. Commun..

[15] Haddock S (2022). Phenotypic and molecular states of IDH1 mutation-induced CD24-positive glioma stem-like cells. Neoplasia (New York, N.Y.).

[16] Alghamri MS (2020). Tumor mutational burden predicts survival in patients with low-grade gliomas expressing mutated IDH1. Neuro-oncol. Adv..

[17] Li S, Lai M, Zhou J, Zhen J, Cai L (2021). PATH-22. Genetic variation between IDH mutant and IDH wild-type glioma. Neuro-oncology.

[18] Cohen A (2015). DNA copy number analysis of Grade II–III and Grade IV gliomas reveals differences in molecular ontogeny including chromothripsis associated with IDH mutation status. Acta Neuropathol. Commun..

[19] Maher EA (2006). Marked genomic differences characterize primary and secondary glioblastoma subtypes and identify two distinct molecular and clinical secondary glioblastoma entities. Can. Res..

[20] Noushmehr H (2010). Identification of a CpG island methylator phenotype that defines a distinct subgroup of glioma. Cancer Cell.

[21] Neftel C (2019). An integrative model of cellular states, plasticity, and genetics for glioblastoma. Cell.

[22] Patel AP (2014). Single-cell RNA-seq highlights intratumoral heterogeneity in primary glioblastoma. Science (New York, N.Y.).

[23] Tirosh I (2016). Single-cell RNA-seq supports a developmental hierarchy in human oligodendroglioma. Nature.

[24] O’Connor SA (2021). Neural G0: a quiescent-like state found in neuroepithelial-derived cells and glioma. Mol. Syst. Biol..

[25] Scialdone A (2015). Computational assignment of cell-cycle stage from single-cell transcriptome data. Methods.

[26] Hou R, Denisenko E, Forrest ARR (2019). scMatch: a single-cell gene expression profile annotation tool using reference datasets. Bioinformatics.

[27] La Manno G (2018). RNA velocity of single cells. Nature.

[28] Bond AM, Ming G-L, Song H (2015). Adult mammalian neural stem cells and neurogenesis: five decades later. Cell Stem Cell.

[29] Gulaia V (2018). Molecular mechanisms governing the stem cell's fate in brain cancer: factors of stemness and quiescence. Front. Cell. Neurosci..

[30] Shi C, Yang X, Bu X, Hou N, Chen P (2017). Alpha B-crystallin promotes the invasion and metastasis of colorectal cancer via epithelial-mesenchymal transition. Biochem. Biophys. Res. Commun..

[31] Tang M (2019). Transcriptomic profiling of neural stem cell differentiation on graphene substrates. Colloids Surf. B Biointerfaces.

[32] Scheel JR, Ray J, Gage FH, Barlow C (2005). Quantitative analysis of gene expression in living adult neural stem cells by gene trapping. Nat. Methods.

[33] Cardano M (2019). Epsins regulate mouse embryonic stem cell exit from pluripotency and neural commitment by controlling notch activation. Stem Cells Int..

[34] Li S (2019). Targeting β2 subunit of Na(+)/K(+)-ATPase induces glioblastoma cell apoptosis through elevation of intracellular Ca(2). Am. J. Cancer Res..

[35] Berger MF (2012). Melanoma genome sequencing reveals frequent PREX2 mutations. Nature.

[36] Wang Y-P, Lei Q-Y (2018). Metabolic recoding of epigenetics in cancer. Cancer Commun..

[37] Nowakowski TJ (2017). Spatiotemporal gene expression trajectories reveal developmental hierarchies of the human cortex. Science (New York, N.Y.).

[38] Marín O, Rubenstein JLR (2001). A long, remarkable journey: tangential migration in the telencephalon. Nat. Rev. Neurosci..

[39] Park NI (2017). ASCL1 reorganizes chromatin to direct neuronal fate and suppress tumorigenicity of glioblastoma stem cells. Cell Stem Cell.

[40] Dirkse A (2019). Stem cell-associated heterogeneity in Glioblastoma results from intrinsic tumor plasticity shaped by the microenvironment. Nat. Commun..

[41] Januchowski R (2016). Increased expression of several collagen genes is associated with drug resistance in ovarian cancer cell lines. J. Cancer.

[42] Le V-M, Lang M-D, Shi W-B, Liu J-W (2016). A collagen-based multicellular tumor spheroid model for evaluation of the efficiency of nanoparticle drug delivery. Artif. Cells Nanomed. Biotechnol..

[43] Egeblad M, Rasch MG, Weaver VM (2010). Dynamic interplay between the collagen scaffold and tumor evolution. Curr. Opin. Cell Biol..

[44] Tejero R (2019). Gene signatures of quiescent glioblastoma cells reveal mesenchymal shift and interactions with niche microenvironment. EBioMedicine.

[45] Assadian S (2012). p53 inhibits angiogenesis by inducing the production of arresten. Can. Res..

[46] Teodoro JG, Evans SK, Green MR (2007). Inhibition of tumor angiogenesis by p53: a new role for the guardian of the genome. J. Mol. Med..

[47] Badur MG (2018). Oncogenic R132 IDH1 mutations limit NADPH for de novo lipogenesis through (D)2-hydroxyglutarate production in fibrosarcoma sells. Cell Rep..

[48] del Pozo MA (2004). Integrins regulate Rac targeting by internalization of membrane domains. Science (New York, N.Y.).

[49] Guan J-L (2004). Integrins, rafts, rac, and rho. Science (New York, N.Y.).

[50] LettieriBarbato D, Vegliante R, Desideri E, Ciriolo MR (2014). Managing lipid metabolism in proliferating cells: new perspective for metformin usage in cancer therapy. Biochim. Biophys. Acta.

[51] Yao CH (2016). Exogenous fatty acids are the preferred source of membrane lipids in proliferating fibroblasts. Cell Chem. Biol..

[52] Baffy G (1858). Mitochondrial uncoupling in cancer cells: liabilities and opportunities. Biochim. Biophys. Acta.

[53] Zhao L (2018). Identification of pharmacological inhibitors of conventional protein secretion. Sci. Rep..

[54] Madan V, Koeffler HP (2020). Differentiation therapy of myeloid leukemia: four decades of development. Haematologica.

[55] Carvalho J (2020). Cell reversal from a differentiated to a stem-like state at cancer initiation. Front. Oncol..

[56] Annovazzi L, Mellai M, Caldera V, Valente G, Schiffer D (2011). SOX2 expression and amplification in gliomas and glioma cell lines. Cancer Genom. Proteom..

[57] Zuk PA (2009). The intracellular distribution of the ES cell totipotent markers OCT4 and Sox2 in adult stem cells differs dramatically according to commercial antibody used. J. Cell. Biochem..

[58] Avilion AA (2003). Multipotent cell lineages in early mouse development depend on SOX2 function. Genes Dev..

[59] Baltus GA (2009). Acetylation of Sox2 induces its nuclear export in embryonic stem cells. Stem Cells.

[60] Picelli S (2014). Full-length RNA-seq from single cells using Smart-seq2. Nat. Protoc..

[61] Pfisterer U (2020). Identification of epilepsy-associated neuronal subtypes and gene expression underlying epileptogenesis. Nat. Commun..

[62] Müller S, Cho A, Liu SJ, Lim DA, Diaz A (2018). CONICS integrates scRNA-seq with DNA sequencing to map gene expression to tumor sub-clones. Bioinformatics.

[63] Starostik MR, Sosina OA, McCoy RC (2020). Single-cell analysis of human embryos reveals diverse patterns of aneuploidy and mosaicism. Genome Res..

[64] Amezquita RA (2020). Orchestrating single-cell analysis with Bioconductor. Nat. Methods.

[65] Hao Y (2021). Integrated analysis of multimodal single-cell data. Cell.

[66] Wolf FA, Angerer P, Theis FJ (2018). SCANPY: large-scale single-cell gene expression data analysis. Genome Biol..

[67] Ritchie ME (2015). limma powers differential expression analyses for RNA-sequencing and microarray studies. Nucleic Acids Res..

[68] Miao Z, Deng K, Wang X, Zhang X (2018). DEsingle for detecting three types of differential expression in single-cell RNA-seq data. Bioinformatics.

[69] Kanehisa M, Goto S (2000). KEGG: kyoto encyclopedia of genes and genomes. Nucleic Acids Res..

[70] Kanehisa M (2019). Toward understanding the origin and evolution of cellular organisms. Protein Sci. Publ. Protein Soc..

[71] Kanehisa M, Furumichi M, Sato Y, Ishiguro-Watanabe M, Tanabe M (2020). KEGG: integrating viruses and cellular organisms. Nucleic Acids Res..

[72] Yu G, He QY (2016). ReactomePA: an R/Bioconductor package for reactome pathway analysis and visualization. Mol. BioSyst..

[73] Cao J (2019). The single-cell transcriptional landscape of mammalian organogenesis. Nature.

[74] Aibar S (2017). SCENIC: single-cell regulatory network inference and clustering. Nat. Methods.

[75] Gao J (2013). Integrative analysis of complex cancer genomics and clinical profiles using the cBioPortal. Sci. Signal..

[76] Cerami E (2012). The cBio cancer genomics portal: an open platform for exploring multidimensional cancer genomics data. Cancer Discov..

