# Author Correction: Single-nucleus transcriptomics of IDH1- and TP53-mutant glioma stem cells displays diversified commitment on invasive cancer progenitors

**DOI:** 10.1038/s41598-023-29355-9

**Published:** 2023-02-07

**Authors:** Valeriia Gulaia, Mikhail Shmelev, Aleksander Romanishin, Nikita Shved, Vladislav Farniev, Nikolay Goncharov, Arthur Biktimirov, Irene Lisa Vargas, Konstantin Khodosevich, Alexander Kagansky, Vadim Kumeiko

**Affiliations:** 1 Institute of Life Sciences and Biomedicine, Medical Center, Far Eastern Federal University, Vladivostok, 690922 Russia; 2 A.V. Zhirmunsky National Scientific Center of Marine Biology, FEB RAS, Vladivostok, 690041 Russia; 3 School of Life Sciences, Immanuel Kant Baltic Federal University, Kaliningrad, 236041 Russia; 4 Biotech Research & Innovation Centre (BRIC), The Faculty of Health and Medical Sciences, University of Copenhagen, 2200 Copenhagen, Denmark

Correction to: *Scientific Reports* https://doi.org/10.1038/s41598-022-23646-3, published online 08 November 2022

The original version of this Article omitted an affiliation for Nikolay Goncharov. The correct affiliations are listed below.

Institute of Life Sciences and Biomedicine, Medical Center, Far Eastern Federal University, Vladivostok, 690922, Russia

A.V. Zhirmunsky National Scientific Center of Marine Biology, FEB RAS, Vladivostok, 690041, Russia

The original Article has been corrected.

