# Supplementary material for: Single-nucleus transcriptomics of IDH1- and TP53-mutant glioma stem cells displays diversified commitment on invasive cancer progenitors
Source: Sci Rep. 2022 Nov 8;12:18975. doi: 10.1038/s41598-022-23646-3 (PMC9643511; doi:10.1038/s41598-022-23646-3)
Supplement: Supplementary file 1 — Supplementary Information. [file 41598_2022_23646_MOESM1_ESM.docx]

*Suppelementary Material*


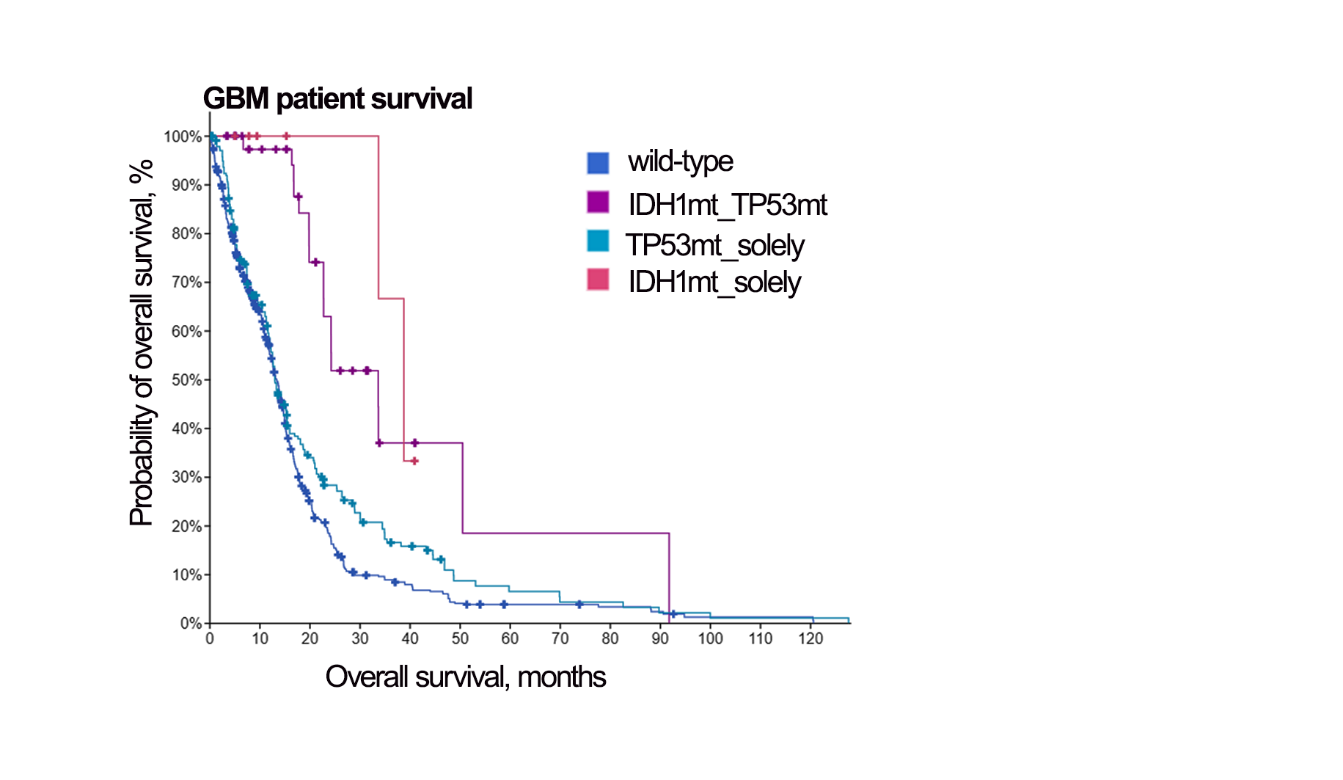


**Figure S1**. **Survival of GBM patients.** The overall survival probability plot of patients with high-grade gliomas depending on mutations in IDH1 and TP53. The color annotated the respective mutational profile – IDH1mut-TP53mis (missense mutations in both IDH1 and TP53 genes), TP53mis_solely (missense mutation only in TP53), IDH1mut_solely (missense mutation only in IDH1), and wild-type (wild-type version of both IDH1 and TP53 genes). Data were taken from the cBioPortal.


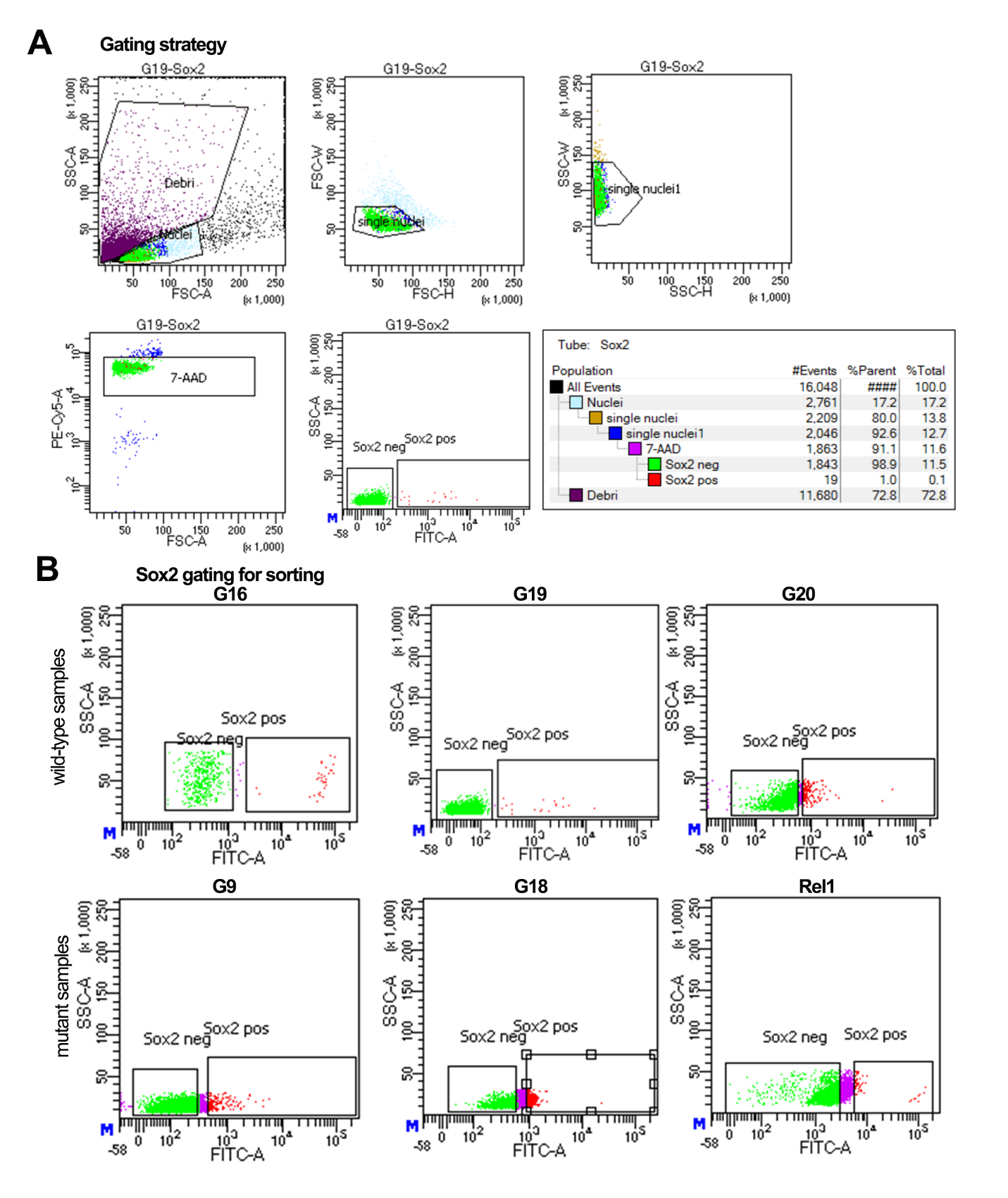


**Figure S2**. **Gating strategy for Sox2+ cell sorting.** (**A**) Representative gating strategy for G19 sample. First gate was to divide nuclei from debri, second and third – to get rid of nuclei dublets, forth gate – to isolate intact nuclei with DNA, fifth gate – to separate Sox2-negative and Sox2-positive population. The position of Sox2-negative gate is based on the isotype IgG-staining with the same secondary antibody. (**B**) Sorting gates for Sox2+ nuclei for all tested samples.

*
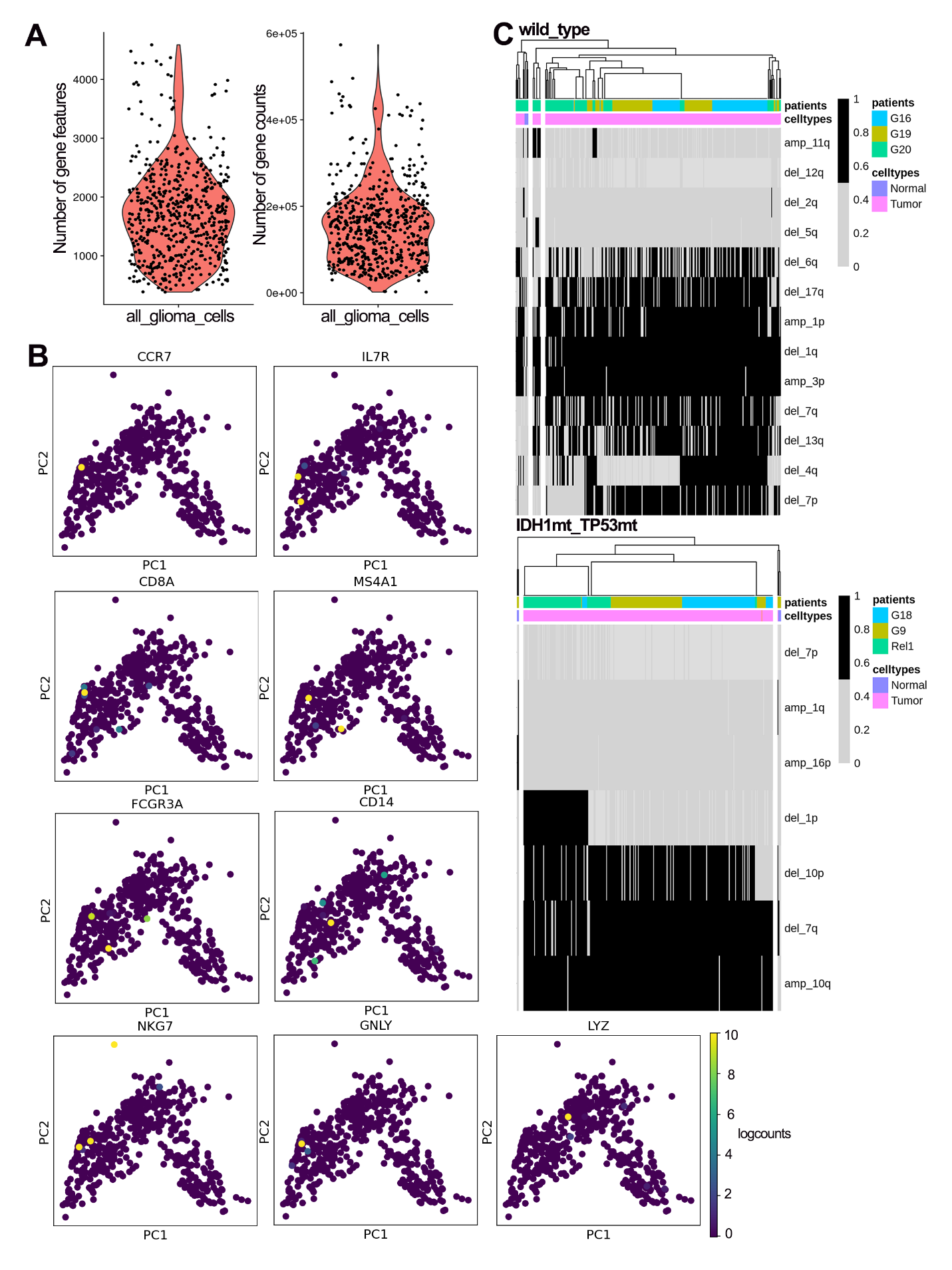
*

**Figure S3**. **Filtering single-cell data.** (**A**) Distribution of the detected gene features and gene counts within all cells supplied for the analysis. (**B**) Expression in logcounts of the hemopoietic genes by all sequenced cells embedded in the PCA plot. (**C**) Binary heatmap of z-scored posterior probabilities for cells (columns) from wt-samples (superior) and mt-samples (inferior) across chromosome regions (rows) indicating the presence (1 – black) or the absence (0 – grey) of the CNV. The first top colour panel annotates cells either as tumor (pink) or normal (violet). The second top panel describe the patient origin of the cells.


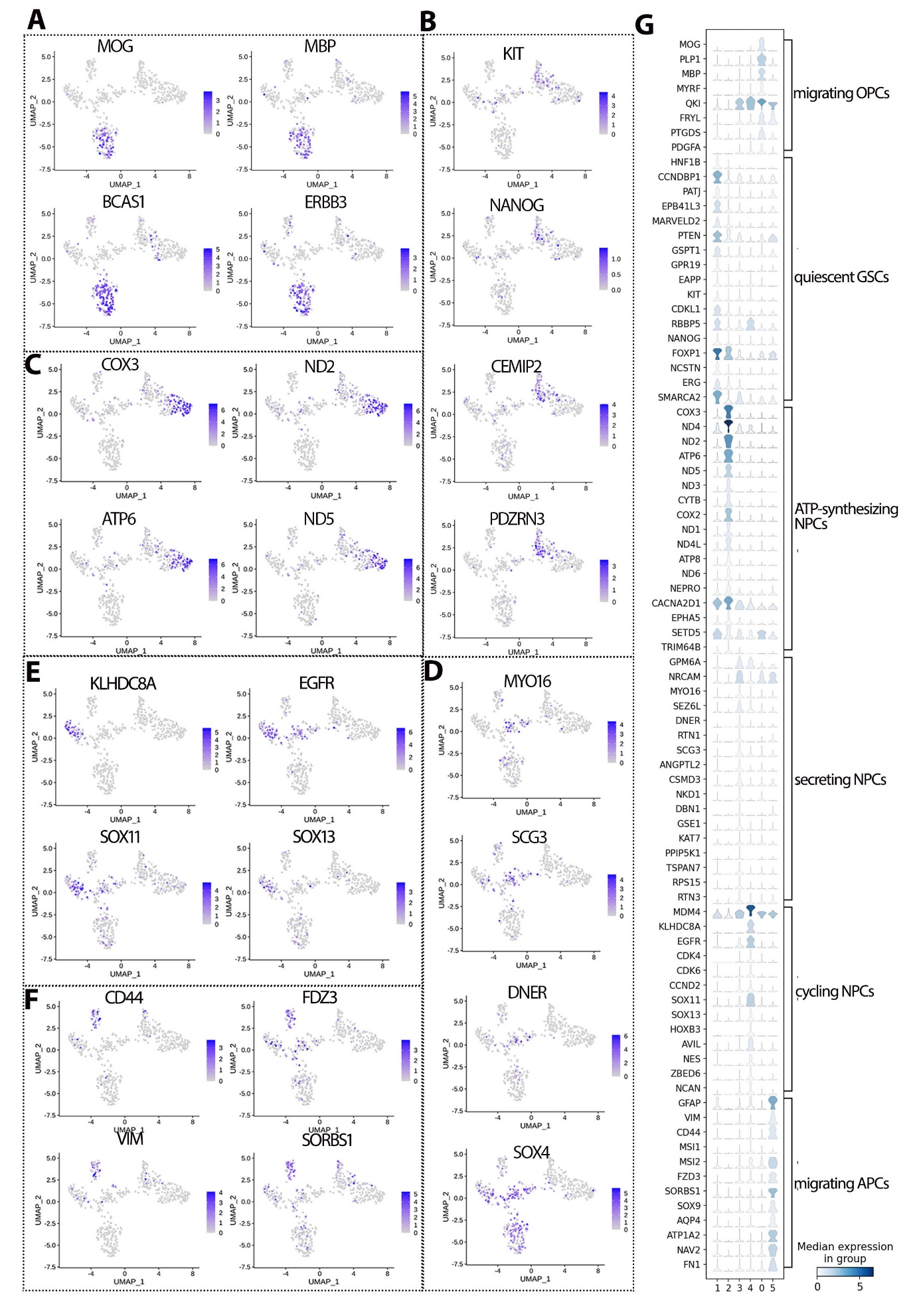


**Figure S4**. **Marker genes for Seurat clusters.** (**A**) distribution of marker genes for cluster 0 on the 2D-UMAP space. (**B**) distribution of marker genes for cluster 1 on the 2D-UMAP space. (**C**) distribution of marker genes for cluster 2 on the 2D-UMAP space. (**D**) distribution of marker genes for cluster 3 on the 2D-UMAP space. (**E**) distribution of marker genes for cluster 4 on the 2D-UMAP space. (**F**) distribution of marker genes for cluster 5 on the 2D-UMAP space. (**G**) Stuck violin plot of marker gene expression for Seurat clusters (bottom panel) and their annotation (right side panel). The violin shape displays the number of the cells expressing a gene, the continuous color panel defines median expression value of a gene from the absence of expression (white) to high expression (dark blue).


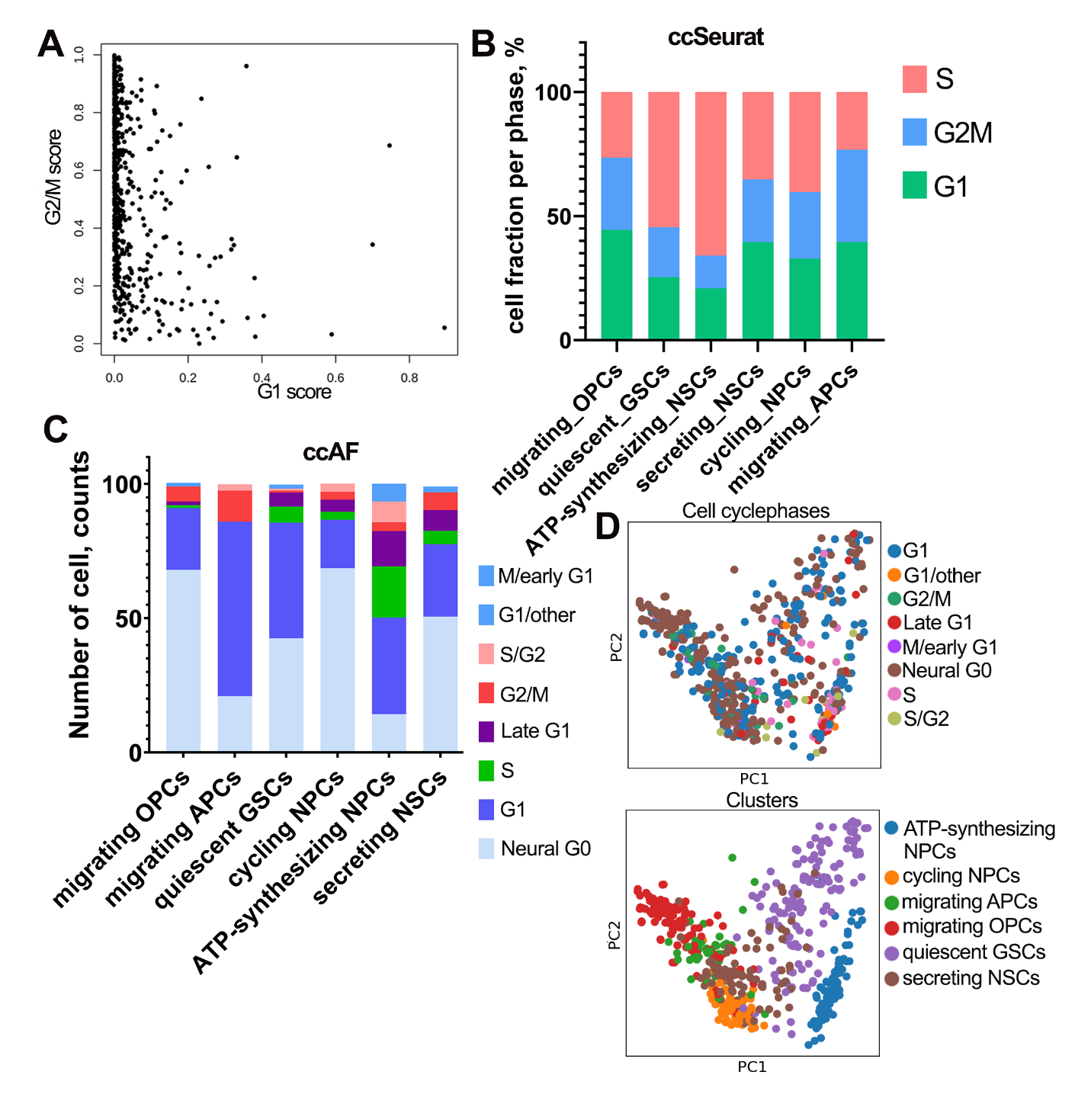


**Figure S5**. **Cell cycle distribution of GSCs.** (**A**) Dotplot represents cells scored on the expression of cell-cycle related genes (G2/M score) versus cells in dormant state (G1 score). G2/M score higher 0.5 means entering S phase of cell cycle. (**B**) Barplot showing the composition of UMAP GSC clusters annotated to cell cycle phases (color bar on the right side) by ccSeurat. (**C**) ccAF annotating UMAP glioma cell clusters to cell-cycle phases described in legend to the right. (**D**) The superior dotplot annotes the distribution of ccAF cell-cycle phases annotated in color dots to the right across Seurat (UMAP) clusters in PCA plots. The inferior dotplot depicts the distribution of Seurat clusters on PCA plot with colorful legend described to the right.


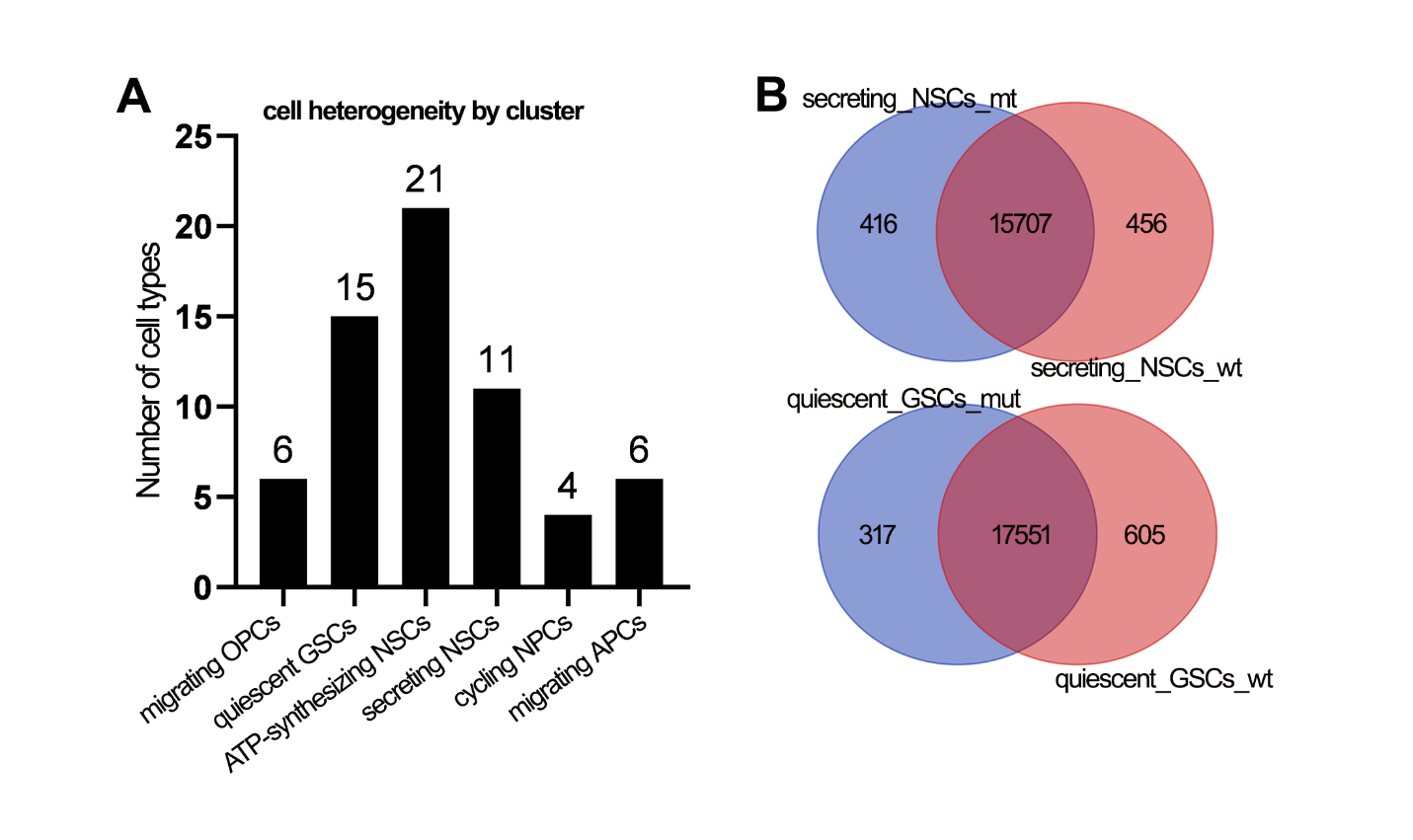


**Figure S6.** **Cell cluster heterogeneity.** (**A**) Bar plot showing the number of cell types assigned by scMatch per Seurat clusters (**B**) Venn diagram annotating the number of unique markers for wt-cells and mt-cells within “secreting NSCs” cluster (superior) and “quiescent GSCs” cluster.


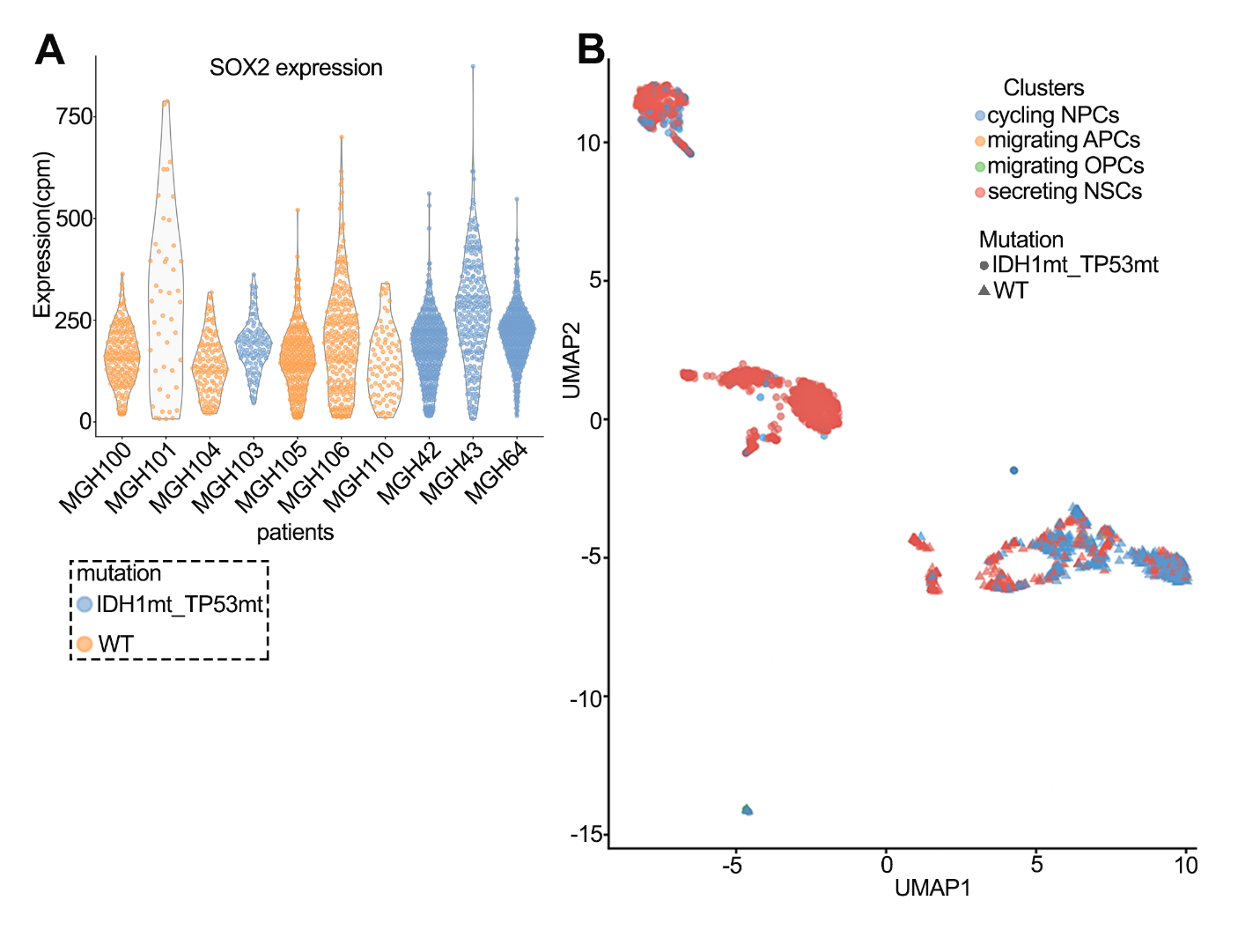


**Figure S7.** **Cluster distribution on different datasets.** (**A**) Sox2 expression by preselected cells from WT (orange) and IDH1mt-TP53mt (blue) datasets. (**B**) UMAP plot of preselected cells from WT (triangle) and IDH1mt-TP53mt (circle) datasets aligned on Seurat clusters from our datasets.


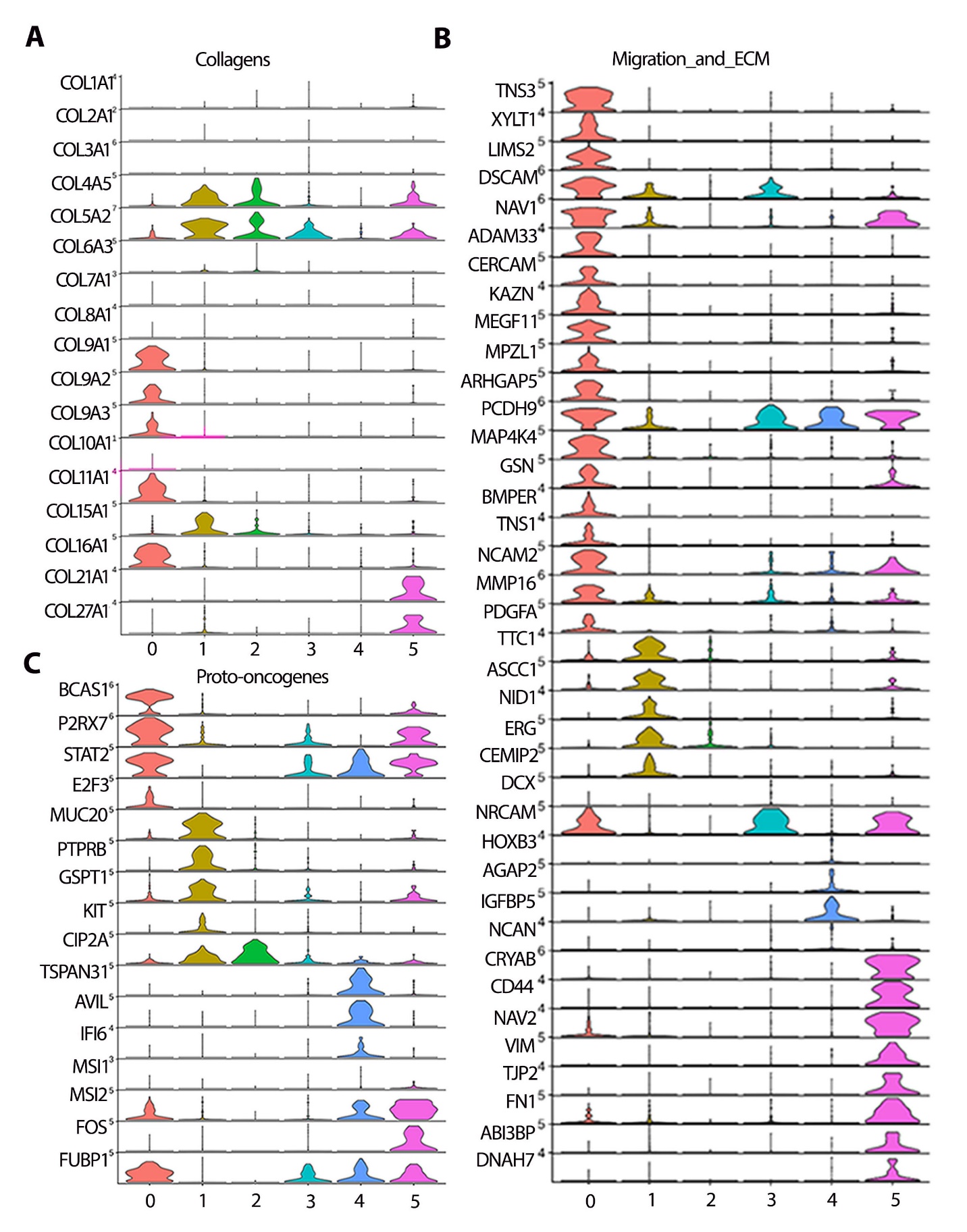


**Figure S8**. **Expression of genes marking cell malignization.** (**A**) expression of collagens in Surat clusters (bottom panel) (**B**) expression of genes linked to Migration and ECM in Surat clusters (bottom panel) (**C**) expression of genes classified as Proto-oncogenes in Surat clusters (bottom panel). The violin shape displays the number of the cells expressing a gene, the violin color defines the Seurat cluster. Gene expression displayed in log-transformed normalized expression values.


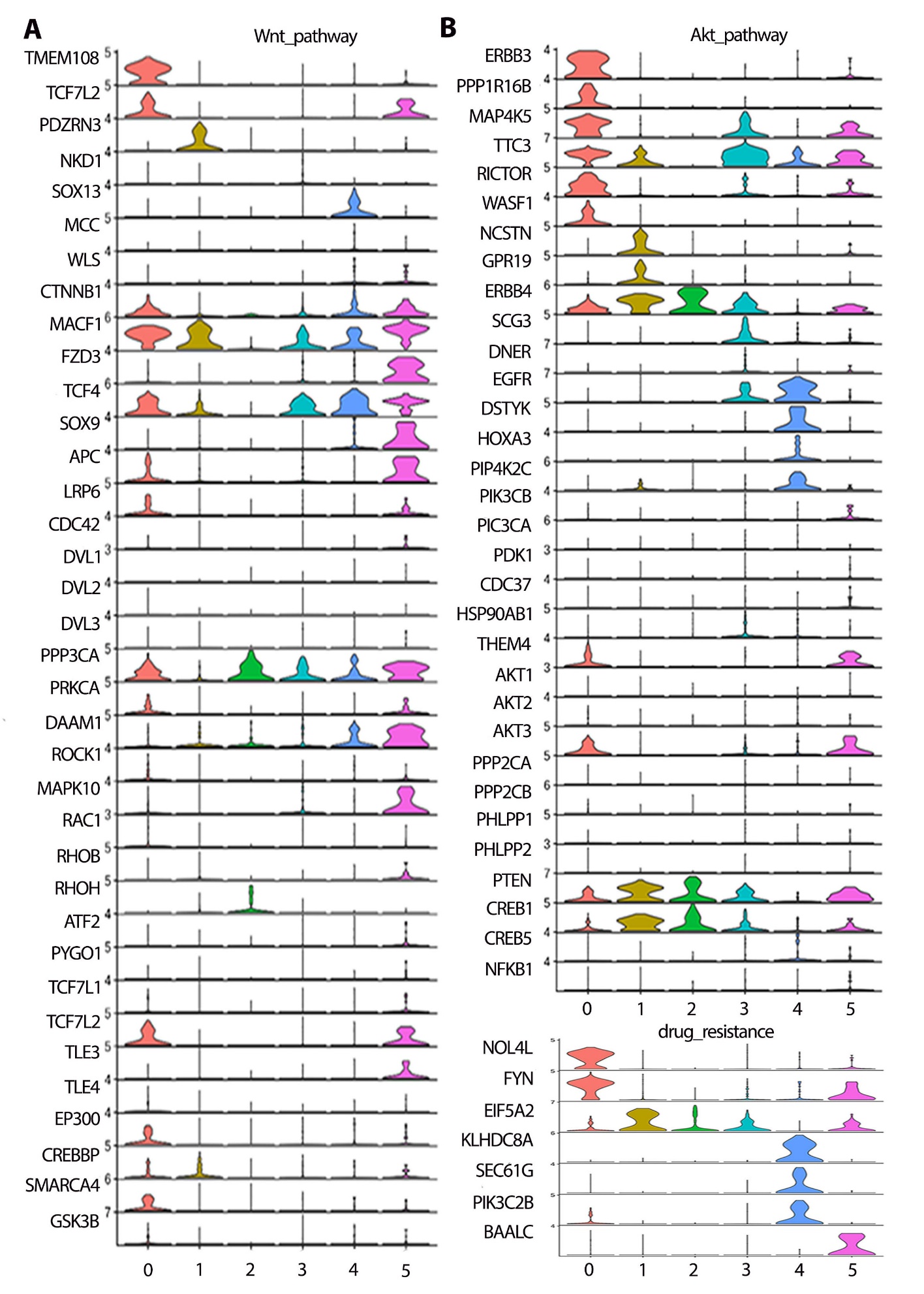


**Figure S9**. **Expression of genes involved in proliferation and survival of cancer cells.** (**A**) Genes involved in Wnt-pathway in Surat clusters (bottom panel). (**B**) Genes involved in Akt-pathway in Surat clusters (bottom panel). (**C**) Genes inducing resistance to cancer therapeutics in Surat clusters (bottom panel). The violin shape displays the number of the cells expressing a gene, the violin color defines the Seurat cluster. Gene expression displayed in log-transformed normalized expression values.


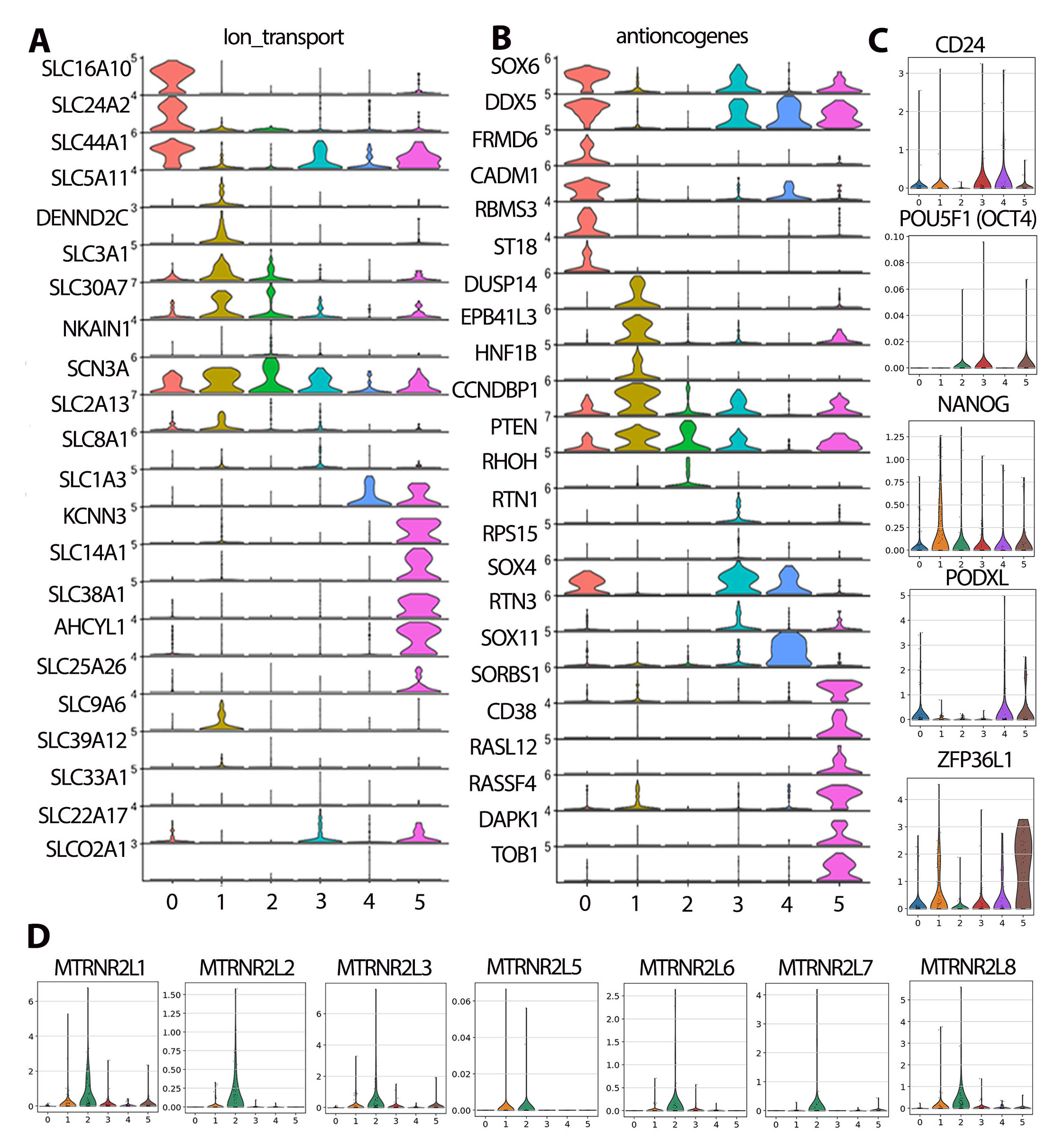


**Figure S10**. **Expression of genes marking CSC profile.** (**A**) Ion channel genes in Surat clusters (bottom panel). (**B**) Antioncogenes in Surat clusters (bottom panel). **C**. Stem-cell genes in Surat clusters (bottom panel). (**D**) Antiapoptotic genes in Surat clusters (bottom panel). The violin shape displays the number of the cells expressing a gene, the violin color defines the Seurat cluster. Gene expression displayed in log-transformed normalized expression values.


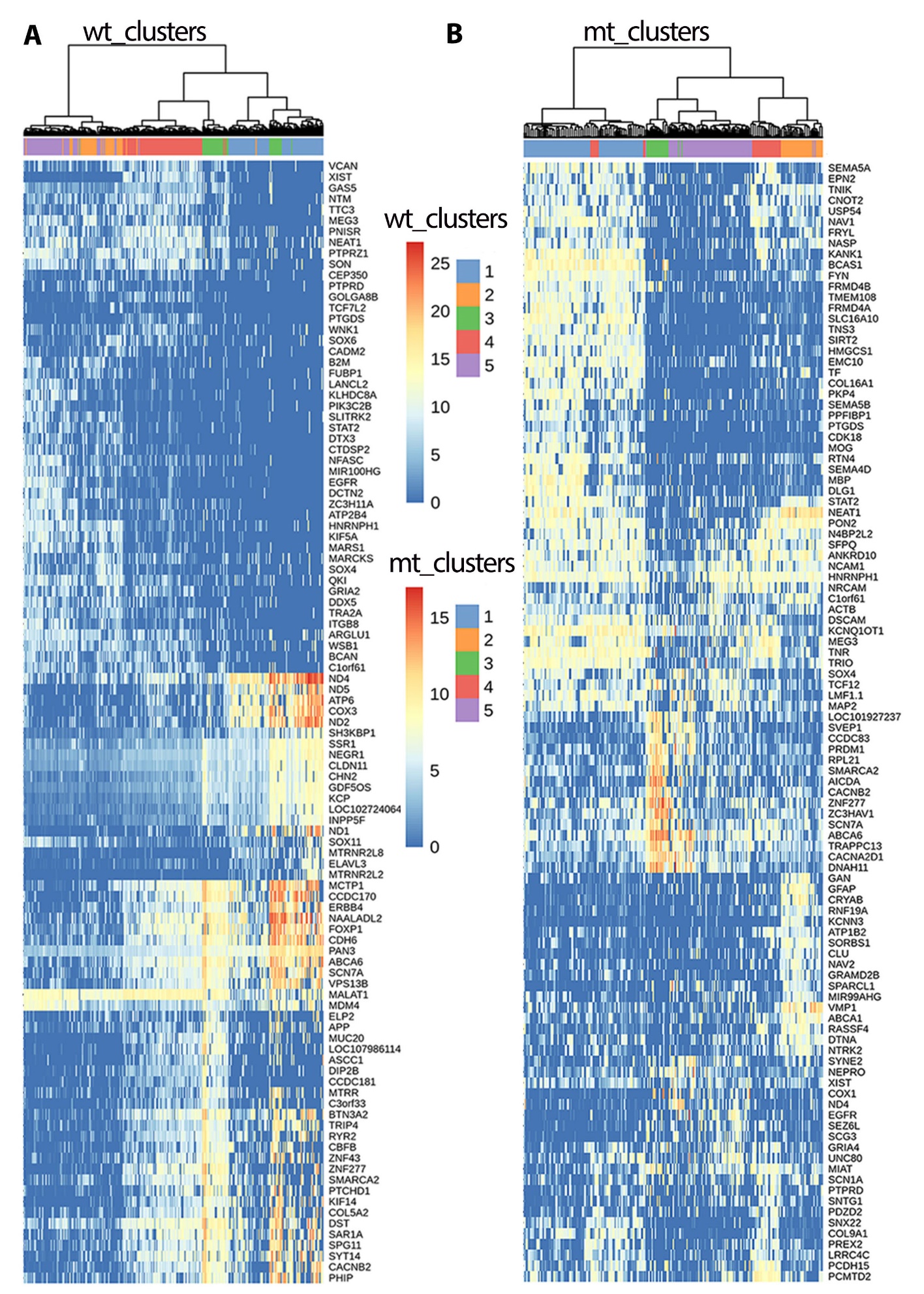


**Figure S11**. **Genes differentially expressed between UMAP clusters**. (**A**) Heatmap for wt-GSCs. Clusters: (1) “ATP-synthesizing NSCs”, (2) “differentiating NPCs”, (3) “migrating NSCs”, (4) “Secreting and migrating NSCs”, (5) “PTPRZ1+ and EGFR+ cycling NPCs”. (**B**) Heatmap for mt-GSCs. Clusters: (1) “migrating OPCs”, (2) “migrating APCs”, (3) “suppressing translation NSCs”, (4) “differentiating NPCs”, (5) “cycling GSCs”. Upper color panel in the heatmap designates Seurat clusters. Gene expression is indicated by continuous color panel starting from the most downregulated (blue) to the most upregulated (red).


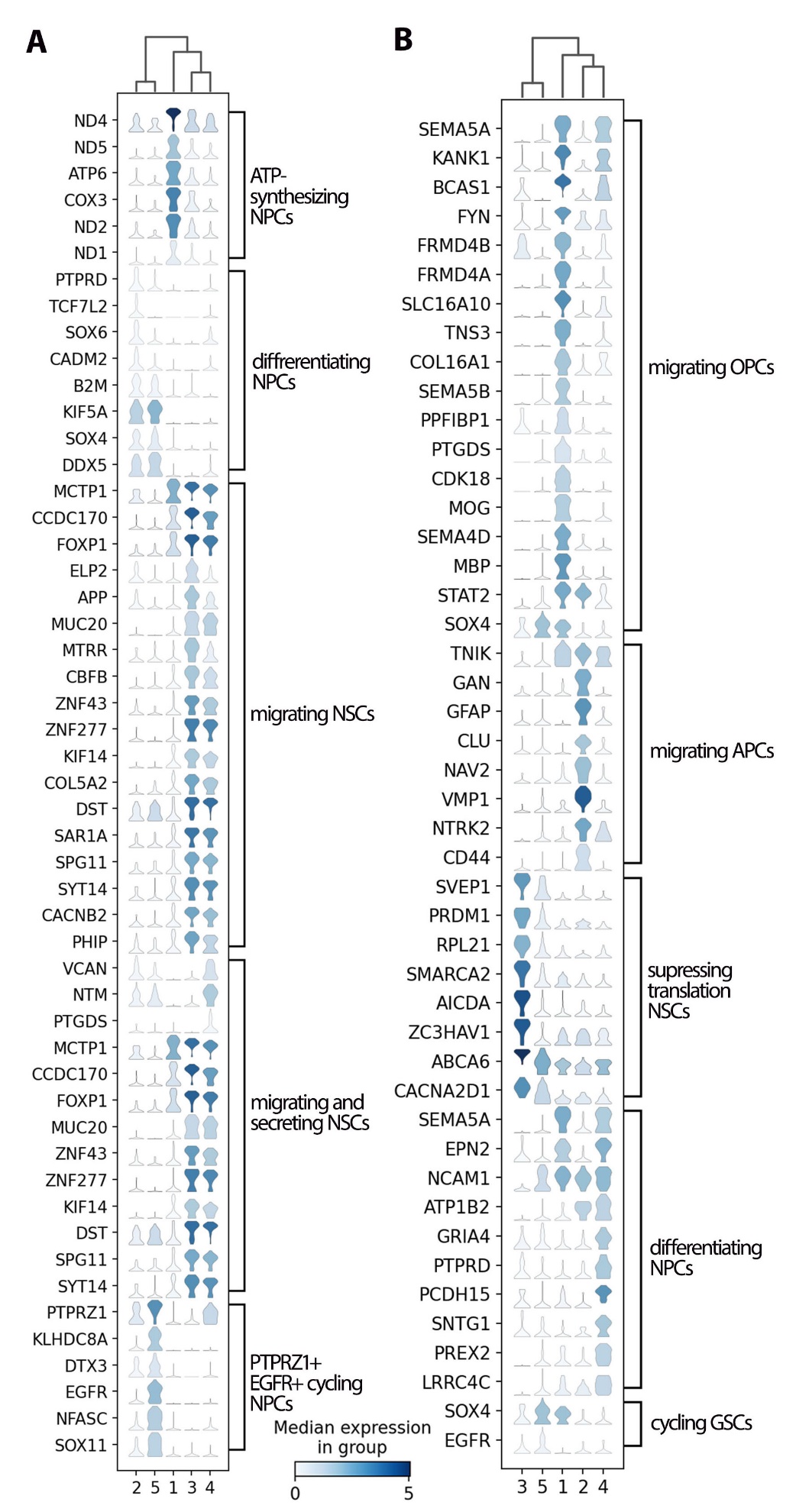


**Figure S12**. **Marker genes defying cell annotations**. (**A**) Stack violin plot displays marker gene expression in wt-GSC clusters. (**B**) Stack violin plot displays marker gene expression in mt-GSC clusters. Genes grouped by cell annotations (side description) and UMAP clusters (down column bar). The violin shape displays the number of the cells expressing a gene, the continuous color panel defines median expression value of a gene from the absence of expression (white) to high expression (dark blue).


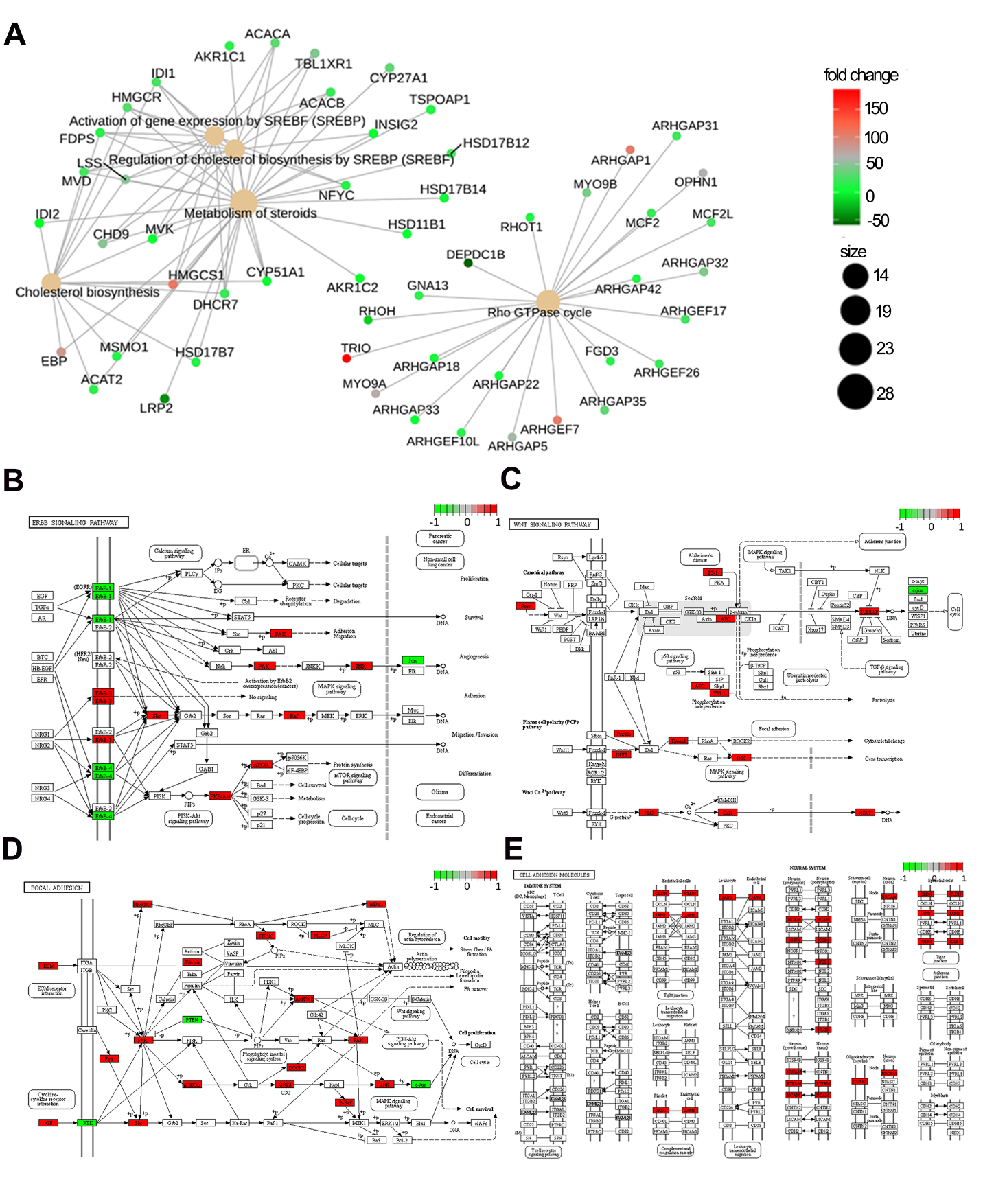


**Figure S13**. **Metabolic and molecular pathways changed in GSCs due to mutations in *IDH1* and *TP53*.** (**A**) Netplot enrichment map of mt-GSCs compared to wt-GSCs visualized using ReactomePA. Big orange dots reflected the changed pathways. Small dots annotated changed genes with the color panel reflecting the fold change level. (**B**) ERBB signaling pathway. (**C**) Wnt signaling pathway. (**D**) Genes linked to Focal adhesion. (**E**) Genes classified as Cell adhesion molecules. Red rectangles display upregulated genes (proteins), green rectangles define downregulated genes (proteins). Pictures obtained by KEGG pathview.


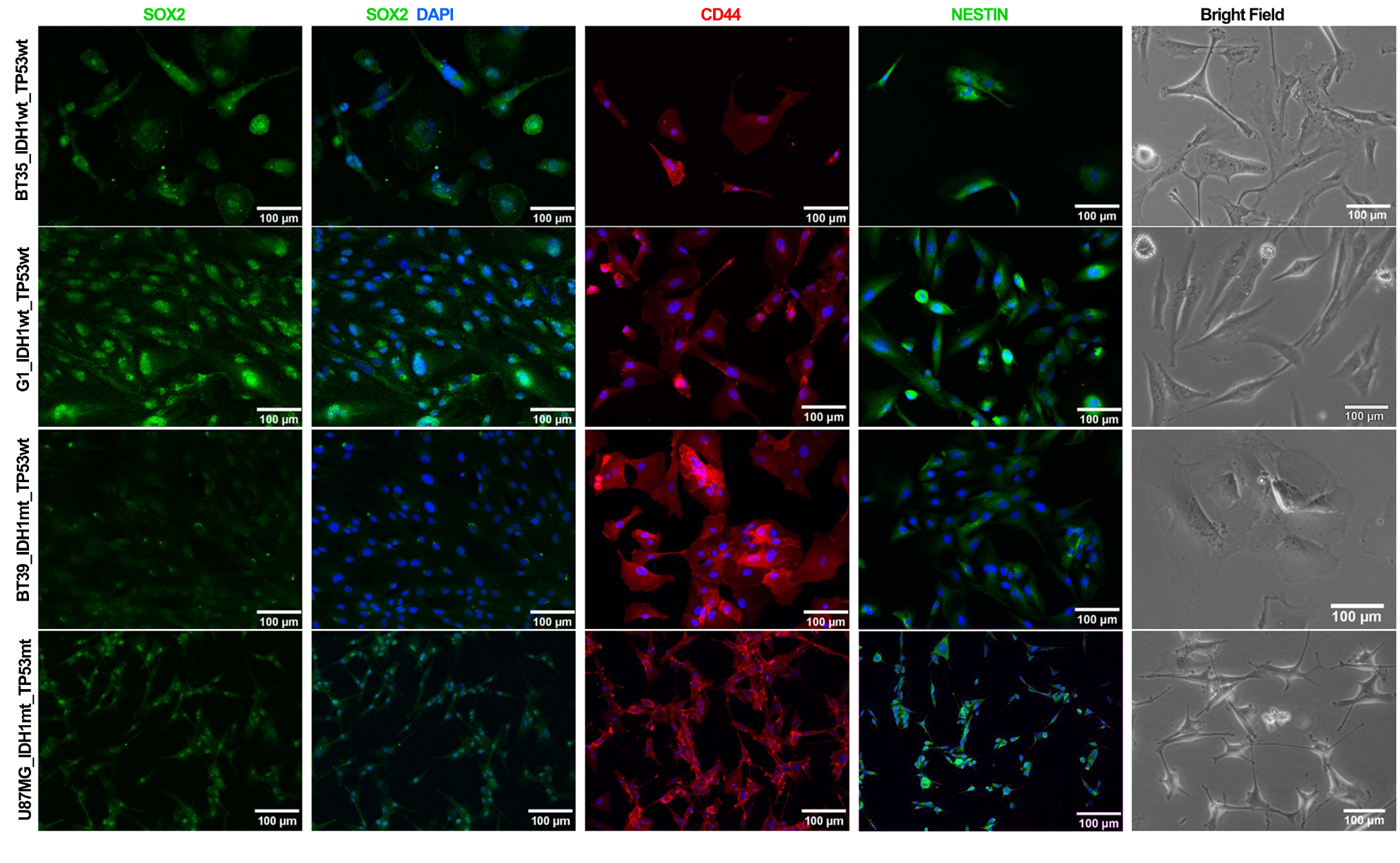


**Figure S14**. **Representative pictures of staining and morphology of human glioma cells.** Staining of glioma cells (IDH1wt_TP53wt cells – superior two rows of pictures, IDH1mt_TP53wt – forth row of pictures, IDH1mt_TP53mt – inferior pictures) for stem (Sox2 and Nestin) and migration (CD44) markers. CD44 displayed in red, Sox2 and Nestin in green, DAPI in blue. The last column depicting cells in Bright Field. All pictures were shot using 10X objective.

**Table S1. Glioma genotyping primers**

| Name | 5’-3’ sequence | Purpose |
| --- | --- | --- |
| IDH1_PCR_Fwd | GTTGAAACAAATGTGGAAATCACC | Amplifying IDH1 DNA fragment for searching substitution at position 395G (R132) |
| IDH1_PCR_Rev | TTCATACCTTGCTTAATGGGTGT |  |
| IDH1_Seq | GTTGAAACAAATGTGGAAATCACC | Sequencing IDH1 DNA fragment for searching substitution at position 395G (R132) |
| IDH2_PCR_Fwd | GCTGCAGTGGGACCACTATTA | Sequencing IDH2 DNA fragment for searching substitution at position 515G (R172) |
| IDH2_PCR_Rev | CAGAGACAAGAGGATGGCTAGG |  |
| IDH2_Seq | CTGTCCTCACAGAGTTCAAGC | Sequencing IDH2 DNA fragment for searching substitution at position 515G (R172) |
| BRAF_PCR_Fwd | CTCTTCATAATGCTTGCTCTG | Amplifying and sequencing BRAF DNA fragment for searching substitution at position 1799T>A (V600E) |
| BRAF_PCR_Rev | GTGAATACTGGGAACTATG |  |
| TP53_R175_F | TTTTGCCAACTGGCCAAGACCT | Amplifying and sequencing TP53 DNA fragment for searching substitution at position 524G (R175) |
| TP53_R175_R | GCCAGACCTAAGAGCAATCAGTG |  |
| TP53_R248_F | GAAACCCCGTCTCTACTGAA | Amplifying and sequencing TP53 DNA fragment for searching substitution at position 742C/743G (R248) |
| TP53_R248_R | GAAGAAATCGGTAAGAGGTGGGC |  |
| TP53_R273_F | GGGAGTAGATGGAGCCTGGTTTTTT | Amplifying and sequencing TP53 DNA fragment for searching substitution at position 817C/818G (R273) |
| TP53_R273_R | GCTTCTTGTCCTGCTTGCTTACC |  |

**Table S2. Smart-seq2 Primers**

| Name | 5’-3’ sequence | Purpose |
| --- | --- | --- |
| Oligo-dT | Biosg/AAGCAGTGGTATCAACGCAGAGTACT30VN | Reverse transcription of mRNA |
| TSO | Biosg/AAGCAGTGGTATCAACGCAGAGTACATrGrGG+ | Switching reaction template from mRNA to cDNA |
| IS PCR | Biosg/AAGCAGTGGTATCAACGCAGAGT | Amplifying cDNA |

**Table S3. LGG/GBM patient overall survival**

| Dataset | Mutations in IDH1 and TP53 | Number of cases, total | Number of events | Median Months Overall (95% CI) |
| --- | --- | --- | --- | --- |
| LGG (low-grade gliomas) | Wild-type | 382 | 124 | 73.42 (51.84 - 105.12) |
|  | IDH1mut_solely | 249 | 35 | 134.27 (95.57 - NA) |
|  | TP53mut_solely | 29 | 12 | 25.46 (19.94 - NA) |
|  | IDH1mut_TP53mut | 367 | 80 | 75.16 (63.55 - 98.23) |
| GBM (glioblastoma) | Wild-type | 723 | 570 | 13.34 (12.46 - 14.19) |
|  | IDH1mut_solely | 7 | 2 | 38.76 (33.70 - NA) |
|  | TP53mut_solely | 242 | 185 | 12.99 (12.23 - 15.40) |
|  | IDH1mut_TP53mut | 46 | 18 | 33.64 (22.72 - NA) |

**Table S4. Description of Seurat clusters**

| Cluster name | Migrating OPCs | Quiescent GSCs | ATP-synthesizing NSCs | Secreting NSCs | Cycling NPCs | Migrating APCs |
| --- | --- | --- | --- | --- | --- | --- |
| Cluster number | Cluster 0 | Cluster 1 | Cluster 2 | Cluster 3 | Cluster 4 | Cluster 5 |
| Number of cell types (scMatch) | 6 | 15 | 21 | 11 | 4 | 6 |
| Number of cells (all) | 144 | 134 | 91 | 91 | 67 | 43 |
| number of wt-cells | 9 | 99 | 91 | 20 | 66 | 3 |
| number of mut-cells | 135 | 35 | 0 | 71 | 1 | 43 |

**Table S5. Information about glioma samples downloaded for additional analysis for Figure S7**

| Study reference | Sample | Patient Diagnosis, Grade | Mutation Status |
| --- | --- | --- | --- |
| GSM3828672 | MGH100 | IDH1 wild-type Glioblastoma, G4 | p53 positive |
| GSM3828672 | MGH101 | IDH1 wild-type Glioblastoma, G4 | - |
| GSM3828672 | MGH104 | IDH1 wild-type Glioblastoma, G4 | mutations in PTEN and TP53 |
| GSM3828672 | MGH105 | IDH1 wild-type Glioblastoma, G4 | EGFR positive, p53 positive |
| GSM3828672 | MGH106 | IDH1 wild-type Glioblastoma, G4 | EGFR positive, p53 positive |
| GSM3828672 | MGH110 | IDH1 wild-type Glioblastoma, G4 | EGFR positive, p53 positive |
| GSE89567 | MGH42 | IDH1 mutant anaplastic astrocytoma, G3 | IDH1 R132H, TP53 R273C, APC S966G |
| GSE89567 | MGH43 | IDH1 mutant anaplastic astrocytoma, recurrent, G3 | IDH1 R132H, TP53 V157F |
| GSE89567 | MGH64 | IDH1 mutant anaplastic astrocytoma, G3 | IDH1 R132H, TP53 R213X and R306X, PIK3CA R88Q |
| GSE89567 | MGH103 | IDH1 mutant anaplastic astrocytoma, G3 | IDH1 R132H, TP53 C299R |

**Table S6. Information about glioma cell lines additionally stained for Sox2, Nestin and CD44 on Supplementary Figure S13**

| Cell line name | Patient Diagnosis, Grade | Patient Gender | Cell Line Mutations |
| --- | --- | --- | --- |
| BT35 | Anaplastic astrocytoma, G3 |  | wild-type |
| G1 | Glioblastoma multiforme, G4 | Female | wild-type |
| BT39 | Anaplastic oligodendroglioma, G2 | Female | IDH1 R132H |
| U87MG | - | - | IDH1 R132H  TP53 R248Q |
